# Vaccine development against tuberculosis before and after Covid-19

**DOI:** 10.3389/fimmu.2023.1273938

**Published:** 2023-11-15

**Authors:** Stefan H. E. Kaufmann

**Affiliations:** ^1^ Max Planck Institute for Infection Biology, Berlin, Germany; ^2^ Systems Immunology, Max Planck Institute for Multidisciplinary Sciences, Göttingen, Germany; ^3^ Hagler Institute for Advanced Study, Texas A&M University, College Station, TX, United States

**Keywords:** tuberculosis, COVID-19, vaccines, correlate of protection, protective antigen, prevention of disease, prevention of infection, prevention of recurrence

## Abstract

Coronavirus disease (Covid-19) has not only shaped awareness of the impact of infectious diseases on global health. It has also provided instructive lessons for better prevention strategies against new and current infectious diseases of major importance. Tuberculosis (TB) is a major current health threat caused by *Mycobacterium tuberculosis* (Mtb) which has claimed more lives than any other pathogen over the last few centuries. Hence, better intervention measures, notably novel vaccines, are urgently needed to accomplish the goal of the World Health Organization to end TB by 2030. This article describes how the research and development of TB vaccines can benefit from recent developments in the Covid-19 vaccine pipeline from research to clinical development and outlines how the field of TB research can pursue its own approaches. It begins with a brief discussion of major vaccine platforms in general terms followed by a short description of the most widely applied Covid-19 vaccines. Next, different vaccination regimes and particular hurdles for TB vaccine research and development are described. This specifically considers the complex immune mechanisms underlying protection and pathology in TB which involve innate as well as acquired immune mechanisms and strongly depend on fine tuning the response. A brief description of the TB vaccine candidates that have entered clinical trials follows. Finally, it discusses how experiences from Covid-19 vaccine research, development, and rollout can and have been applied to the TB vaccine pipeline, emphasizing similarities and dissimilarities.

## 1 Introduction

The Severe Acquired Respiratory Syndrome Corona Virus 2 (SARS-CoV-2) has had an unprecedented impact on our understanding and awareness of the continuous threat of emerging infectious diseases. Coronavirus disease 2019 (Covid-19), caused the death of more than seven million individuals (1, 2) The World Health Organization (WHO) has estimated mortality rates to be approximately 15 million deaths over three years (3). In addition, Covid-19 led to a sudden increase in incidences of numerous other communicable and non-communicable diseases (4). One disease that was been profoundly affected is tuberculosis (TB) (5). This was due to multiple factors, notably the disruption of laboratory services, shortages of drug supply, and deviation of funding and personnel to diagnosis and care of Covid-19 patients. During the height of the Covid-19 crisis, TB morbidity and mortality increased for the first time in the 21st century to 10-11 million new cases and 1.6 million deaths in 2021 (5). It has been estimated that over the last 200 years, TB has been the cause of one billion deaths averaging annual mortality in the order of five million deaths over 200 years, similar to the estimated five million annual deaths caused by Covid-19 over three years (5–9). It is now clear that TB patients after successful therapy can develop post-TB, which not only affects the lungs but can also lead to other disabilities, notably neurological and cardiac impairments. The Covid-19 crisis led to reduced case findings and therapy for TB. This coincidence will likely lead to the increased appearance of post-TB. Thus, the consequences of the Covid-19 TB syndemic will have a much greater impact on health and consequently on economic losses in the years to come (10, 11). Yet, to my knowledge, there is no direct information regarding the impact of Covid-19 on post-TB.

As soon as the pandemic potential of SARS-CoV-2 became apparent, multiple efforts were undertaken to develop and deploy Covid-19 vaccines at unprecedented speed (6). Research and development (R&D) of Covid-19 vaccines could build on knowledge gathered in the aftermath of the emergence of SARS-CoV-1 and the Middle East Respiratory Syndrome (MERS) virus, even though these coronaviruses had been brought under control by conventional public health measures (12). Through these efforts, it became clear that the Spike protein mediates virus attachment to and entry into host cells and that blocking the viral attachment by neutralizing antibodies represents a key protective mechanism (13).

Supported by virtually unlimited funding, the research and development (R&D) of SARS-CoV-2 vaccines was pursued at accelerated speed through remarkable collaborations between scientific communities across continents (14). Furthermore, adoptive trial design, streamlined regulatory processes, expedited regulatory review and rapid emergency use approval made vaccine rollout possible within less than one year (15). Complemented by scaled-up manufacturing capacities, millions of lives could be saved. The mRNA encapsulated in lipid nanoparticles (LNP) turned out the most efficacious vaccine platform (16). Although this platform was a new aspect of the vaccine portfolio, its manufacturing could be scaled up rapidly. In total, more than 13 billion doses were deployed in record time, nearly fulfilling the demands of the industrialized world. This scenario, however, was overshadowed by inequitable access to vaccines in low- and middle-income countries (17).

Vaccine R&D in general has benefited from the example of the Covid-19 vaccine pipeline in several instances. First, virtually unlimited investment into novel vaccines in the very beginning does not only save lives but also generates a return on investment (14, 18, 19). A study in New York provides an illustrative example by showing that 10 US$ was saved for every 1 US$ invested in vaccination against Covid-19 (19). Second, adoptive trials combining safety and efficacy assessments are feasible (20). Third, accelerated regulatory processes as well as provisional authorization for emergency use act as accelerators. Fourth, vaccine rollout at a large scale in high-income countries proved that logistic, manufacturing, and deployment hurdles can be overcome. Fifth, the speed of vaccine development can be markedly accelerated by sharing data and samples. Sixth, vaccines need to be made available across continents including low- and middle-income countries (17). As a corollary, R&D centers as well as manufacturing facilities, complemented by an infrastructure that guarantees adequate education, trust, and expertise in the global south are needed to ensure a robust supply chain for equitable access to vaccines (17, 21).

Applying the lessons learned in recent vaccine R&D will enable a more rapid response against future emerging diseases with pandemic potential. This will also promote vaccine R&D against diseases that already pose an enormous threat and for which efficacious vaccines are not yet available, such as Human Immunodeficiency Virus/Acquired Immunodeficiency Syndrome (HIV/AIDS), malaria, Hepatitis C, Dengue, and TB. There will be no strategy that fits all; thus, specific modifications are critical for each vaccination strategy under development. It is also unclear whether the new mRNA : LNP vaccine type, which was so successful in the case of Covid-19, can be applied to infectious diseases that are chronic and controlled by complex cell-mediated rather than humoral immunity, such as TB.

This article briefly discusses the major vaccine platforms in general terms (section 2), summarizes the major Covid-19 vaccines (section 3), and reviews immunity in TB (section 4). It then discusses different vaccination regimes and hurdles for vaccine R&D relevant to TB (section 5) before describing the pipeline of TB vaccines in clinical trials in more detail (section 6). The final sections examine which lessons from Covid-19 vaccine R&D could benefit the TB vaccine portfolio and what approach TB research needs to undertake (sections 7 and 8).

## 2 Major vaccine platforms

Vaccines can be divided into subunit vaccines or whole-cell vaccines (22). To ensure induction of adequate immunity, major subunit vaccine platforms comprise: (i) well-defined antigen(s) formulated in adjuvant, (ii) mRNA encoding such antigen(s) and packaged in LNP (mRNA : LNP), or (iii) bacterial or viral vectors expressing such antigen(s). Whole-cell vaccines are either inactivated non-viable or attenuated viable vaccines. They more or less comprise all antigens of the pathogen independent of their role in protective immunity.

### 2.1 Subunit vaccines

The most successful subunit vaccines target pathogens that are primarily controlled by neutralizing antibodies (22). These types of vaccines depend on one or a few protective antigen(s) that either cause disease directly or are critical for the establishment of stable infection, e.g. by mediating entry into host cells. These include antiviral vaccines (e.g. Hepatitis B), antitoxin vaccines (diphtheria and tetanus), or conjugate vaccines (pneumococci). Further improvement can be accomplished by generating virus-like particles in which the protective antigen forms structured particles resembling the viral pathogen. First-generation adjuvants, notably aluminum salts primarily stimulate the production of neutralizing antibodies.

More recent advances have led to the creation of adjuvant formulations that also stimulate cell-mediated immune responses including CD4 and CD8 T cells (23–26). These novel adjuvants include surface-active components such as saponins (e.g. QS21, an active compound from the bark of Quillaja Saponaria), ligands for pattern recognition receptors, notably toll-like receptors (TLRs), and aqueous and oleaginous formulations that ensure continuous antigen release over prolonged periods of time. The choice of TLR ligands depends on the type of pathogen targeted by the specific vaccine, e.g. TLR-7/TLR-8 ligands for viral and TLR-9 ligands for bacterial pathogens. Examples of T- cell stimulating adjuvants are AS01_E_ (adjuvant system 01_E_) and ISCOM (immune stimulating complex) based adjuvants (23–25). Alternatively, recombinant viral vectors expressing vaccine antigen(s) have been generated, which are mostly replication-deficient (27–31). The recently licensed Ebola vaccination scheme is based on a prime/boost scheme comprising adenovirus (Ad) 26 and Modified Vaccinia Ankara (MVA) virus as vectors, both expressing Ebola antigen (32). Another example is the chimpanzee adenovirus Oxford (ChAdOx) vector expressing the Spike protein of SARS-CoV-2 against Covid-19 (Vaxzevria by Oxford/AstraZeneca).

The major breakthrough in mRNA : LNP vaccine development was the encoding of modified Spike protein (mRNA: LNP) (16, 33, 34). These vaccines exploit Methyl-Pseudouridine modifications of mRNA leading to superior vaccine efficacy compared to unmodified mRNA. The higher efficacy of modified mRNA over unmodified mRNA is likely due to the more rapid inactivation by an innate immune response (35). Principally, LNP are composed of long-chain fatty acids, cholesterol, and polyethylene glycol. The latter may be substituted by polysarcosine with a lower risk of adverse events. In short, LNP (i) protect RNA from rapid degradation; (ii) facilitate introduction into host cells; and (iii) provide a certain degree of adjuvanticity.

### 2.2 Whole cell vaccines

Whole cell vaccines are preferred when protective antigens do not exist or have not been identified. They are given in inactivated form or as attenuated live vaccines. Several inactivated vaccines have been successfully deployed for viral infections such as the inactivated vaccines against Hepatitis A, Polio (Salk vaccine), and Influenza. Yet, only a few inactivated vaccines have been introduced for control of bacterial infections such as the Cholera vaccine. To improve the protective immune response, adjuvants may be required. In contrast, attenuated viable vaccines generally get by without adjuvant. Attenuated vaccines have been most successfully deployed against viral pathogens including measles, mumps, rubella, or polio (Sabin vaccine). The most widely distributed attenuated vaccine against a bacterial pathogen, Bacille-Calmette-Guérin (BCG), targets TB, but with limited success (36).

## 3 A short primer on Covid-19 vaccines

**Figure 1:**
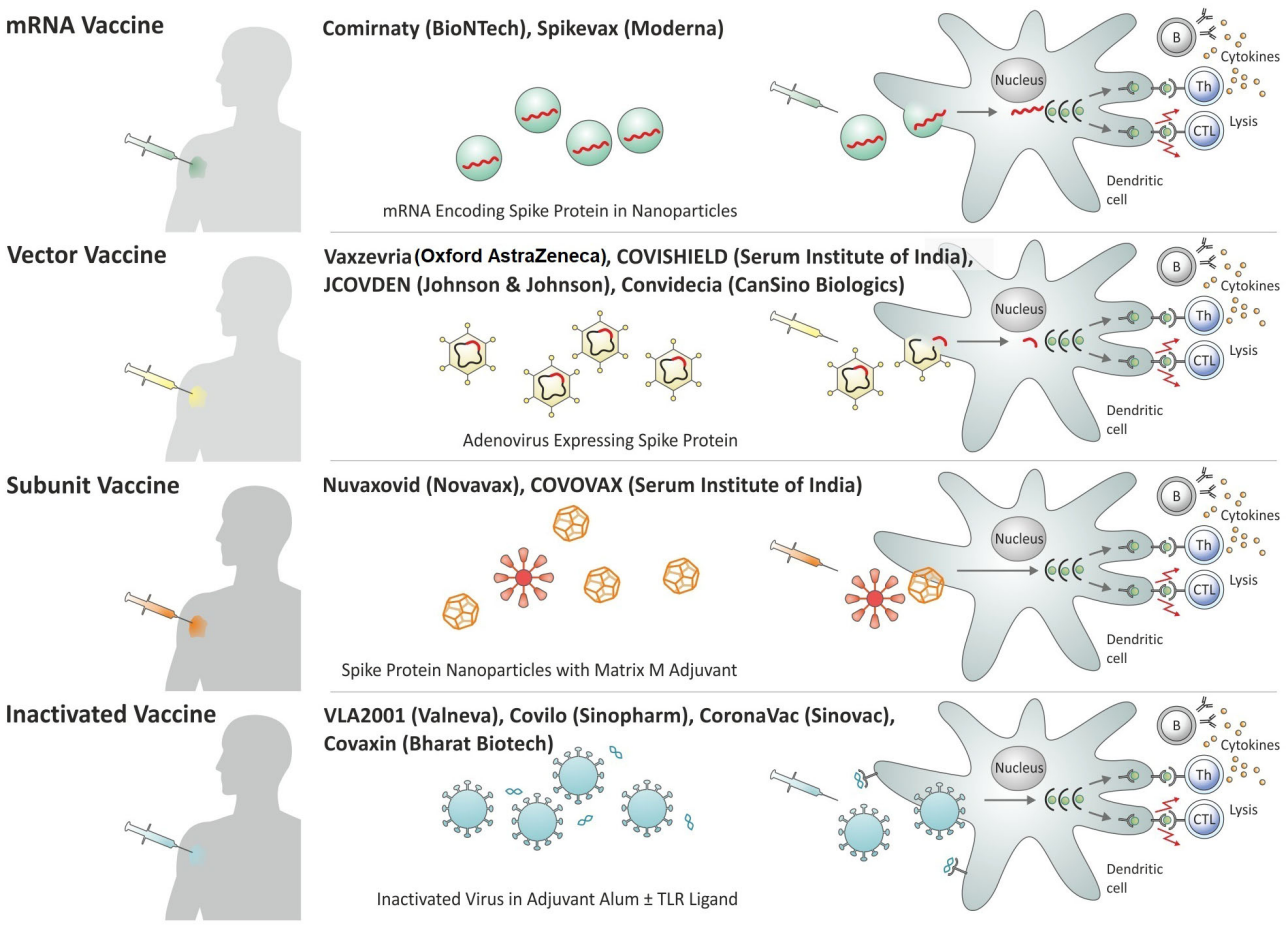
Overview of Covid-19 vaccines granted Emergency Use Listing (EUL) by the World Health Organization (WHO) by platform.

Roughly one year after the introduction of the first vaccines against Covid-19, numerous vaccines had been rolled out in different regions of the globe and more than 11 vaccines have been granted emergency use listing (EUL) by the WHO (37). These include inactivated whole cell vaccines, viral-vectored vaccines, protein:adjuvant vaccines, and mRNA : LNP vaccines. Some of these vaccines had been approved in a large number of states, notably for emergency use; others had received approval in only a few countries. In the following, a brief description of the most widely used vaccines granted EUL by the WHO is provided (**Figure 1**).

The inactivated whole cell vaccines Covilo and CoronaVac by Sinopharm or Sinovac, respectively, had been approved rapidly in China and received permission for emergency use in other countries (38, 39). Both vaccines had been inactivated with beta-propiolactone and formulated in alum salt as adjuvant to stimulate neutralizing antibodies (40). These vaccines probably possess low T cell stimulatory activity because of the exclusive use of alum and they would likely benefit from a T cell stimulating adjuvant. In contrast, the inactivated vaccine VLA2001 of Valneva is formulated in alum salt plus CpG as a TLR-9 agonist, thereby stimulating both humoral and cellular immune responses (41). Similarly, Covaxin from Bharat Biotech contains inactivated SARS-CoV-2 formulated in alum adsorbed TLR-7/TLR-8 agonist (42).

Ad has been the preferred vector system for the expression of the Spike protein. These include human Ad26 and Ad5 as well as the chimpanzee Ad, ChAdOx (30). These Ad serotypes were chosen to avoid rapid inactivation of the carrier by pre-existing antibodies induced by circulating Ad serotypes. The prevalence of Ad26 and Ad5 in humans is low and ChAdOx does not circulate in humans. To avoid the generation of novel virus particles in the immunized host, the Ad vectors have been rendered non-replicative. The ChAdOx vaccine (Vaxzevria from Oxford AstraZeneca, COVISHIELD from Serum Institute of India) given as homologous prime/boost, has been broadly deployed (42, 43). The Ad26-based vaccine JCOVDEN from Janssen (Johnson & Johnson) and the Ad5-based vaccine Convidecia from CanSino Biologics are considered single shot vaccines (44, 45).

The protein:adjuvant vaccine (Nuvaxovid from Novavax, COVOVAX from Serum Institute of India) had received emergency use in several countries (40, 46). This vaccine is composed of protein nanoparticles (similar to virus-like particles) incorporated in the Matrix-M adjuvant containing saponin and based on ISCOM.

Within less than a year, the mRNA : LNP vaccines turned out to be most efficacious with the frontrunners produced by Pfizer/BioNTech (Comirnaty) and Moderna (Spikevax) (34, 47, 48). The mRNA : LNP vaccines comprise a modified mRNA encoding part of the Spike protein as an antigen. The mRNA : LNP vaccines do not only stimulate neutralizing antibodies but also T cell responses that recognize conserved epitopes in the Spike protein, which are broadly shared with various coronaviruses including circulating viruses and novel variants of SARS-CoV-2. Hence, they elicit protective immunity against severe disease even in cases in which the highly specific antibodies fail to adequately neutralize new mutations in the Spike protein.

In summary, the major lessons learned from the R&D of the Covid-19 vaccines can be summarized as follows:

- Neutralizing antibodies specific for the receptor binding domain (RBD) within the Spike protein directed at the angiotensin converting enzyme 2 (ACE-2) receptor reduce infection by blocking attachment to and entry into host cells of SARS-CoV2 (49). Because of the intracellular lifestyle of Mtb, which primarily resides in macrophages, neutralizing antibodies against protective antigens do not exist in TB (see 4). This represents the Achilles’ heel of TB vaccine development. These neutralizing antibodies are highly specific and hence cause immune pressure favoring viral mutations to evade protective immunity. Selection of such mutated strains can rapidly lead to the emergence and spreading of novel strains which render available vaccines partially ineffective.
- Aside from neutralizing antibodies, non-neutralizing antibodies, and T lymphocytes specific for epitopes located outside of the RBD of the Spike protein are being generated (50–52). Non-neutralizing antibodies contribute to protection via additional effector mechanisms, notably complement activation, attraction of inflammatory cells, and arming of NK cells for antibody-dependent cellular cytotoxicity (ADCC).
- T lymphocytes directed at conserved epitopes in the Spike protein can contribute to protection at later stages, notably through lysis of infected cells, which ultimately blocks viral replication (53, 54). Aside from these direct effector functions, mostly executed by CD8 T cells with cytolytic activity (cytolytic T lymphocytes, CTL), CD4 helper T cells (Th cells) are activated. Neutralizing antibodies depend on Th2 cells, whereas non-neutralizing antibodies require help from both Th1 and Th2 cells. Th1 cells are also required for activation of CTL and mononuclear phagocytes and Th17 cells can attract inflammatory cells to the site of viral replication.

The development of effective Covid-19 vaccines was an outstanding success story. Yet, in the long-term, a universal pan-corona vaccine providing long-term protection would be extremely valuable. Such next generation vaccines should induce an immune response comprising:

- neutralizing antibodies to the Spike RBD;
- trained immunity for rapid dampening of infection (55);
- broadly reactive antibodies for conserved epitopes with low selection advantage (50, 51, 56);
- CD4 and CD8 T cells to conserved Spike epitopes and perhaps other viral components with low selection advantage;
- additionally, unconventional T cells such as mucosal-associated invariant T (MAIT) cells should be considered.

**Figure 2:**
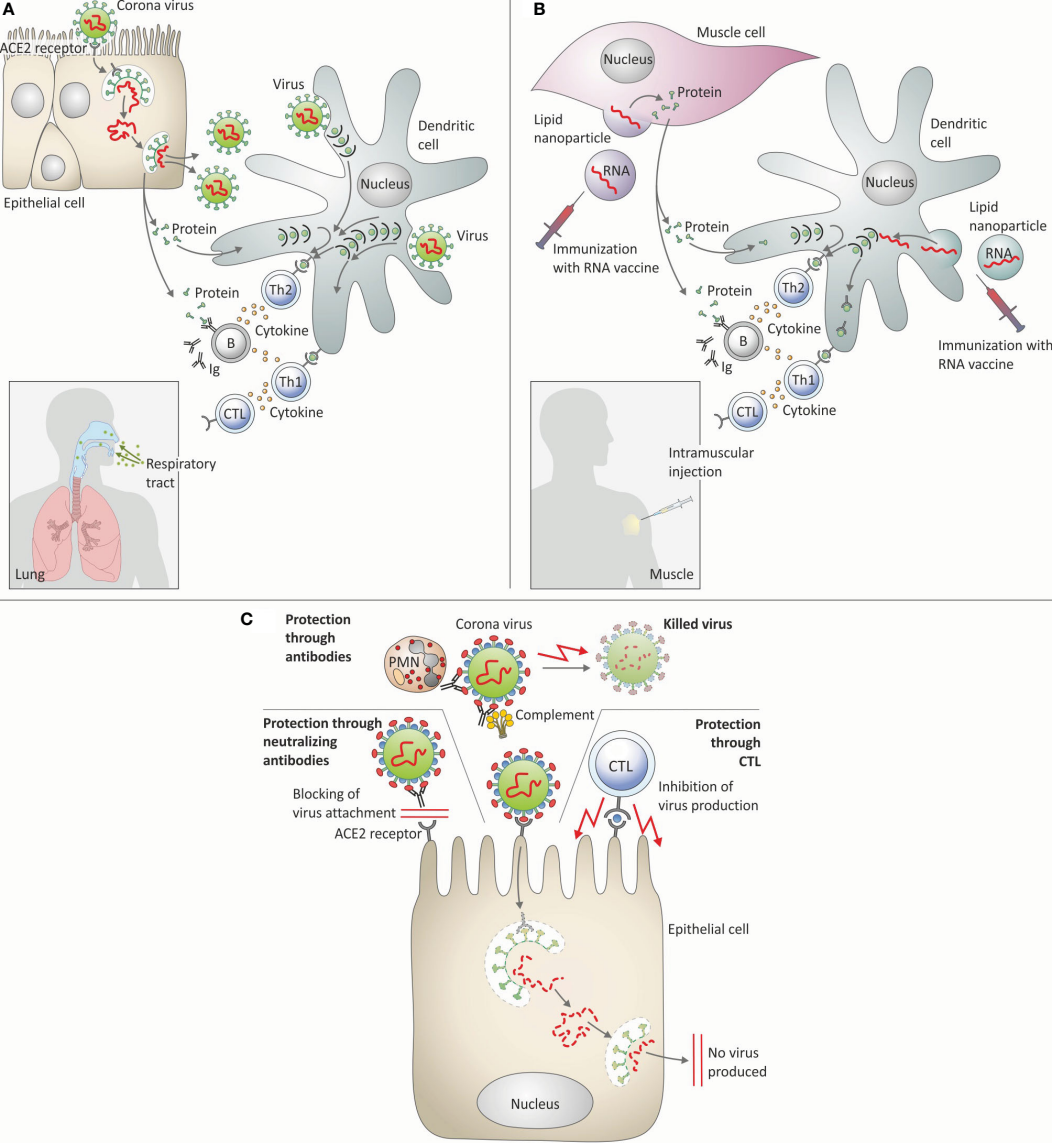
Immune response elicited by SARS-CoV-2 infection and by mRNA : LNP vaccination. **(A)** Infection with SARS-CoV-2. **(B)** Immunization with mRNA: LNP. **(C)** Protective immune response against SARS-Cov-2. ACE-2 receptor, Angiotensin converting enzyme 2 receptor; B, B cells; CTL, Cytolytic T lymphocytes; Ig, Immunoglobulin; PMN, Polymorphonuclear neutrophils; Th1, T helper 1 cells; Th2, T helper 2 cells.

**Figure 2** schematically summarizes protective immunity elicited by SARS-CoV-2 infection and by mRNA : LNP vaccines against Covid-19.

## 4 Immunity in TB: protection and pathology

**Figure 3:**
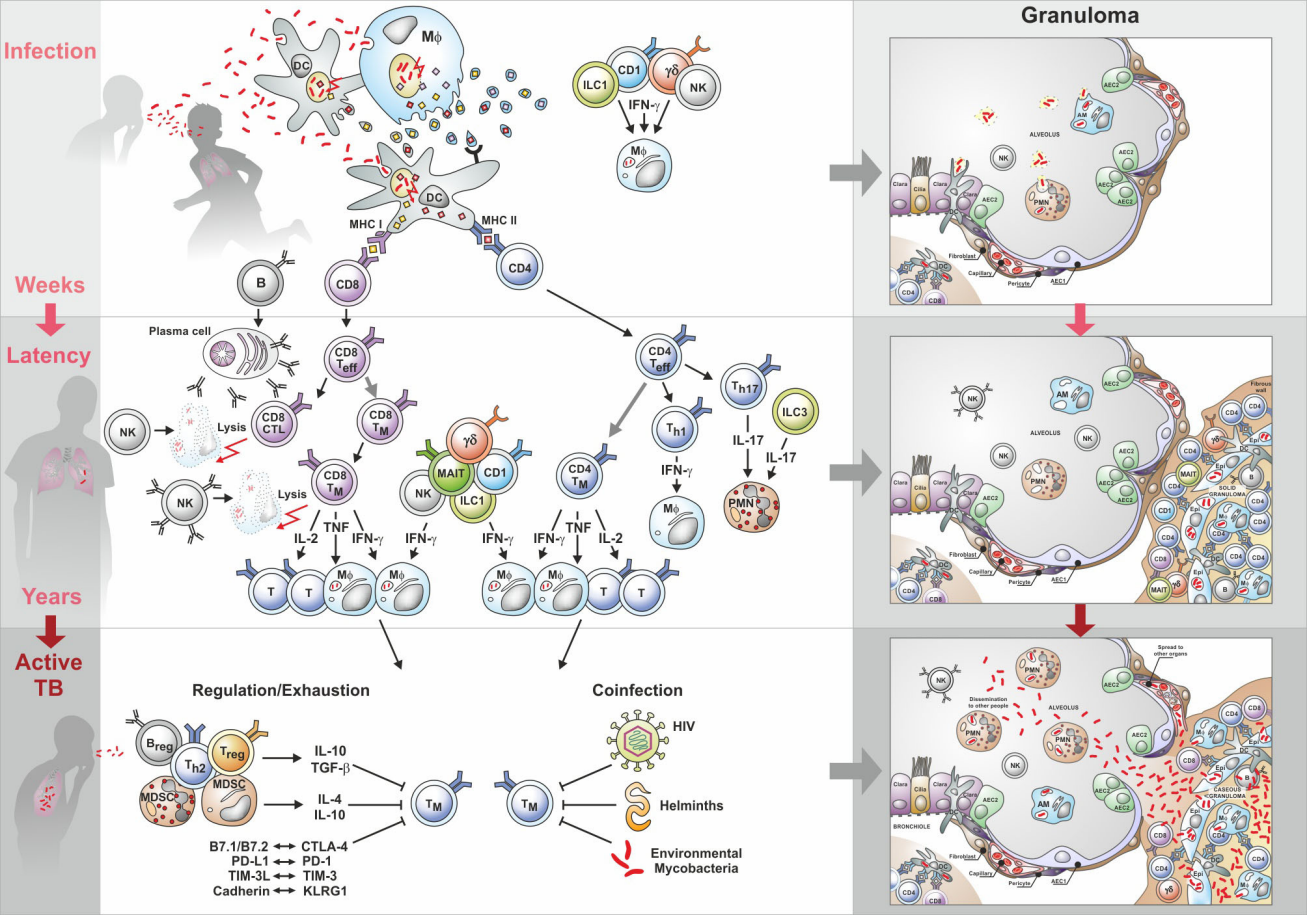
Immunity to tuberculosis (TB): from infection to active disease. Upper part shows cell interactions induced by infection with Mtb; the middle section describes interactions during latency; the lower part describes cell interactions underlying active TB. Characteristic granuloma stages are depicted on the right side. AEC, Alveolar epithelial cells; AM, Alveolar macrophages; B, B cells; CD4, CD4 T helper cells; CD8, CD8 T helper cells; CTL, Cytotoxic T lymphocytes; CTLA-4, Cytotoxic T-lymphocyte-associated protein-4; Epi, Epithelial cell; γ/δ, Gamma/delta cells; IFN-γ, Interferon-γ; ILC, Innate lymphoid cells; IL, Interleukin; KLRG1, Killer cell lectin-like receptor G1; MAIT, Mucosal-associated invariant T cells; MDSC, Myeloid derived suppressor cells; MHC, Major histocompatibility complex; Mφ, Macrophage; NK, Natural killer cells; PD-1, Programmed cell death protein 1; PD1-L, Ligand for PD1; PMN. Polymorphonuclear neutrophils; T, T cells; Teff, T effector cells; Treg, T regulatory cells; TGF, Transforming growth factor; Th1, T helper 1 cells; Th2, T helper 2 cells; Th17, T helper 17 cells; TIM-3, T-cell immunoglobulin and mucin domain-containing protein 3; TIM-3L, Ligand for T-cell immunoglobulin and mucin domain-containing protein 3; TM, Memory T cells; TNF, Tumor necrosis factor.

Establishment of infection (**Figure 3** ): TB is primarily a disease of the lung that also serves as the main port of entry for the causative agent, *Mycobacterium tuberculosis* (Mtb) (57, 58). TB is transmitted via aerosols, coughed up by a patient with active TB although other modes of transmission are possible. Pathogens transmitted via the aerogenic route enter the lung alveoli within small aerosol particles which provide some shield for Mtb. Bacteria are engulfed by alveolar macrophages, tissue-resident mononuclear phagocytes with the capacity for self-renewal. In addition, notably after the onset of inflammation and attracted by chemokines and other attractants, neutrophils, and monocytes enter alveoli from the blood circulation, which are capable of engulfing Mtb (59). The pathogen is transported to different sites of the lung parenchyma by mononuclear phagocytes. At the site of Mtb deposition, granulomas begin to develop independently from each other (60, 61). Some Mtb may be killed by the phagocytes soon after infection, notably in individuals who have been immunized with BCG and/or carry latent TB infection (LTBI). In this situation, macrophages could develop trained immunity based on epigenetic changes (62). Evidence for the participation of natural killer (NK) cells in early defense against Mtb has been presented (63). These NK cells are rapidly attracted to the Mtb-infected lung. It is a matter of discussion whether early infection control can lead to sterile eradication in so-called non-converters (see 5.1).

Initiation of the acquired immune response (**Figure 3** ): Interstitial dendritic cells (DC) transport Mtb to draining lymph nodes and chemokines attract additional DC as well as T lymphocytes and B lymphocytes into specialized structures in the lymph node, where the acquired immune response is activated (64–66). CD4 T cells of Th1 type, which produce multiple cytokines, are considered of critical importance (67). The role of CD4 T cells in controlling TB is probably best illustrated by the aggravated outcome of HIV-Mtb coinfection (68). HIV impairs CD4 T cells and people living with HIV (PLWH) are highly susceptible to TB (69). Interferon-γ (IFN-γ) and tumor necrosis factor α (TNFα) are of major importance as they activate mononuclear phagocytes directly (70, 71). The critical role of TNFα in controlling Mtb in infected individuals became obvious when patients with rheumatoid arthritis treated with anti-TNF monoclonal antibodies frequently progressed to active TB (72). IL-2 could contribute to protection by activating other lymphocyte subsets, notably CD8 T cells. In addition to Th1 cells, also Th17 producing cells are considered important, notably at the early stage of infection (73, 74). CD4 T cells are restricted by the major histocompatibility complex class II (MHC II) and hence are primarily focused on macrophages and DC. CD8 T lymphocytes contribute to protection via the secretion of IFN-γ and TNFα. In addition, they also directly attack infected host cells by means of perforin and granzyme. Moreover, human CD8 T cells produce granulysin which has been shown to directly kill Mtb (75–77). Because of their MHC I restriction, CD8 T cells possess a much broader target spectrum than CD4 T cells (78, 79). Hence, they monitor virtually all nucleated cells, e.g. epithelial cells surrounding alveoli, which can harbor Mtb.

Unconventional T cells are potential contributors to defense against Mtb including (80, 81):

- γ/δ T cells which recognize so-called phospho-antigens and have been found to produce IL-17 (74, 80, 82, 83);
- CD1-restricted T cells which recognize glycolipids prevalent in Mtb and are potent cytokine producers (84);
- MAIT cells which recognize non-peptide antigens and are prevalent in the respiratory tract (74, 78, 85, 86).

These populations are considered donor-unrestricted since they recognize non-peptidic epitopes in the context of unconventional presentation molecules that lack the heterogeneity of canonical MHC molecules that restrict conventional T cell responses (87). The innate lymphoid cells (ILC) are characterized by the absence of T cell receptor (TCR) and hence do not recognize antigens at all (88, 89). They are present in mucosal surfaces and at tissue sites such as the lung and likely participate in the early defense against Mtb. Similar to Th lymphocytes, ILC segregates into subtypes according to their cytokine profile. Hence, by producing IFN-γ and TNFα or IL-17, ILC contributes to protective immunity in TB, notably during the early stages. NK cells can be viewed as ILC since they lack the TCR and are of lymphoid origin (63). Yet, they are not tissue resident and circulate through the blood stream. In conclusion, the role of conventional T cells in TB is well accepted, whereas the participation of unconventional T cells and ILC remains less well understood.

B lymphocytes first play a role in TB by regulating immune responses, mostly by means of cytokines (90, 91). Second, they are the cellular source of antibodies (92). Antibodies can support protective immunity by facilitating phagocytosis, formation of phagosome/lysosome fusion, and stimulation of reactive oxygen and nitrogen intermediates (92–96). Indeed, evidence had already been presented in the 1970s that antibodies mediating the uptake of Mtb through the FcR promote phagosome/lysosome fusion for bactericidal activities (97, 98). Another role of antibodies in TB is the arming of NK cells. Evidence has been presented that NK cells can kill infected cells via ADCC (63).

Immunity during LTBI (**Figure 3** ): The description of the different cell populations should not be interpreted to mean that these cells act independently; rather, they crosstalk with each other and it is this complex interplay between the different cells of the innate and acquired arm of immunity and their secretion products (notably cytokines, chemokines, and antibodies), which results in protective immunity capable of containing Mtb and thus preventing progression to active TB (99–102). At the risk of oversimplification, fine-tuned immunity controls the infection and at the same time keeps inflammation at a minimum. This is the case with LTBI which affects one quarter of the world population. Maladapted immunity fails to control infection and inflammation, thereby allowing progression to active TB (**Figure 3**). This complex immune response is highly sensitive to perturbations. Notably, in the absence of correlates of protection (see 5.5.2), the mechanisms underlying effective host control in 90% of individuals infected with Mtb and progression to active TB disease in 10% of these remain elusive. Notably, it is unclear whether this failure is due to exhaustion or active downregulation of the protective immune components (**Figure 3**). Obviously, efficacious vaccines against TB need to induce a fine-tuned immune balance (58, 103, 104).

Because Mtb interferes with the buildup of protective immunity, it takes several weeks before granulomatous lesions develop into solid granulomas that contain Mtb. Within these granulomas, different populations of Mtb-specific lymphocytes, mononuclear phagocytes, DC, and other cell types exist in a well-organized structure. T lymphocytes will develop into memory T cells, which segregate into effector memory T cells, central memory T cells, and resident memory T cells (105–107). Resident memory T cells seem to be of particular importance (108). Active granulomas can induce the formation of lymphoid follicles in their vicinity, which participate in the orchestration of the solid granuloma (99, 109, 110).

Progression to necrotic and caseous granulomas (**Figure 3** ): A maladapted immune response promotes the transition of solid granulomas to necrotic and then caseous granulomas (59, 103, 104). This progression from LTBI to active TB disease can occur months to years after infection. The maladaptation may be caused by exogenous factors such as coinfection with HIV or helminths or through endogenous factors, which can be summarized as suppression and exhaustion. The latter mechanisms are still incompletely understood. It is likely that suppressive mononuclear phagocytes, the myeloid derived suppressor cells (MDSC), regulatory B cells, and regulatory T cells contribute to the transition into necrotic/caseous granulomas (90, 111–113). Moreover, evidence has been presented for the role of checkpoint control in TB, e.g. through interactions between programmed cell death protein 1 (PD-1) and ligand for PD-1 (PDL-1) or between T-cell immunoglobulin and mucin domain-containing protein 3 (TIM-3) and ligand of TIM-3 (TIM-3L) (114, 115). Evidence suggests that blocking checkpoint control in TB causes excessive immunity characterized by elevated TNF-α production further emphasizing the importance of fine-tuned immunity in TB control and of maladapted immunity as a critical factor of active TB disease (116). The deteriorating immune response in the granulomas causes marked cell destruction, leading to loss of structure and function. In parallel, the lack of granuloma structure allows access of Mtb to capillaries that facilitate transmission to other organs in the body and to alveoli, which promote spread into the environment. At this stage, patients suffer from active TB and are contagious.

During their residence in the solid granuloma, Mtb organisms are mostly in a dormant stage, i.e. they show low to absent metabolic and replicative activity (64, 117, 118). Once the immune response worsens and caseous granulomas develop Mtb ‘wakes up’ and transits into a replicative and metabolic active stage. The cellular detritus in the caseous granuloma favors Mtb growth. The switch from dormant to active Mtb could have consequences for the selection of vaccine antigens (117, 119). Vaccines targeting the prevention of infection (PoI) in naïve individuals could benefit from active Mtb antigens, whereas vaccines targeting the prevention of disease (PoD) in LTBI would exploit dormant Mtb antigens. Consistent with this, a preponderance of dormancy associated antigens has been identified in individuals with LTBI (120).

Vaccines for both PoI (reinfection) and PoD in individuals with LTBI will require both types of antigens.

The granuloma landscape in the lung is heterogeneous (61, 99). During the early stages of LTBI, lesions of different developmental stages coexist which will then mature into solid granulomas (121). During progression to active TB, granulomas transit into the necrotic and then caseous stage. Hence, during incipient/pre-clinical TB, necrotic lesions emerge. This seems to induce an increased inflammatory response which can be determined in the blood by means of transcriptomic and metabolomic biosignatures, which can be harnessed for prognosis of active TB (122–126).

## 5 Vaccination strategies

**Figure 4:**
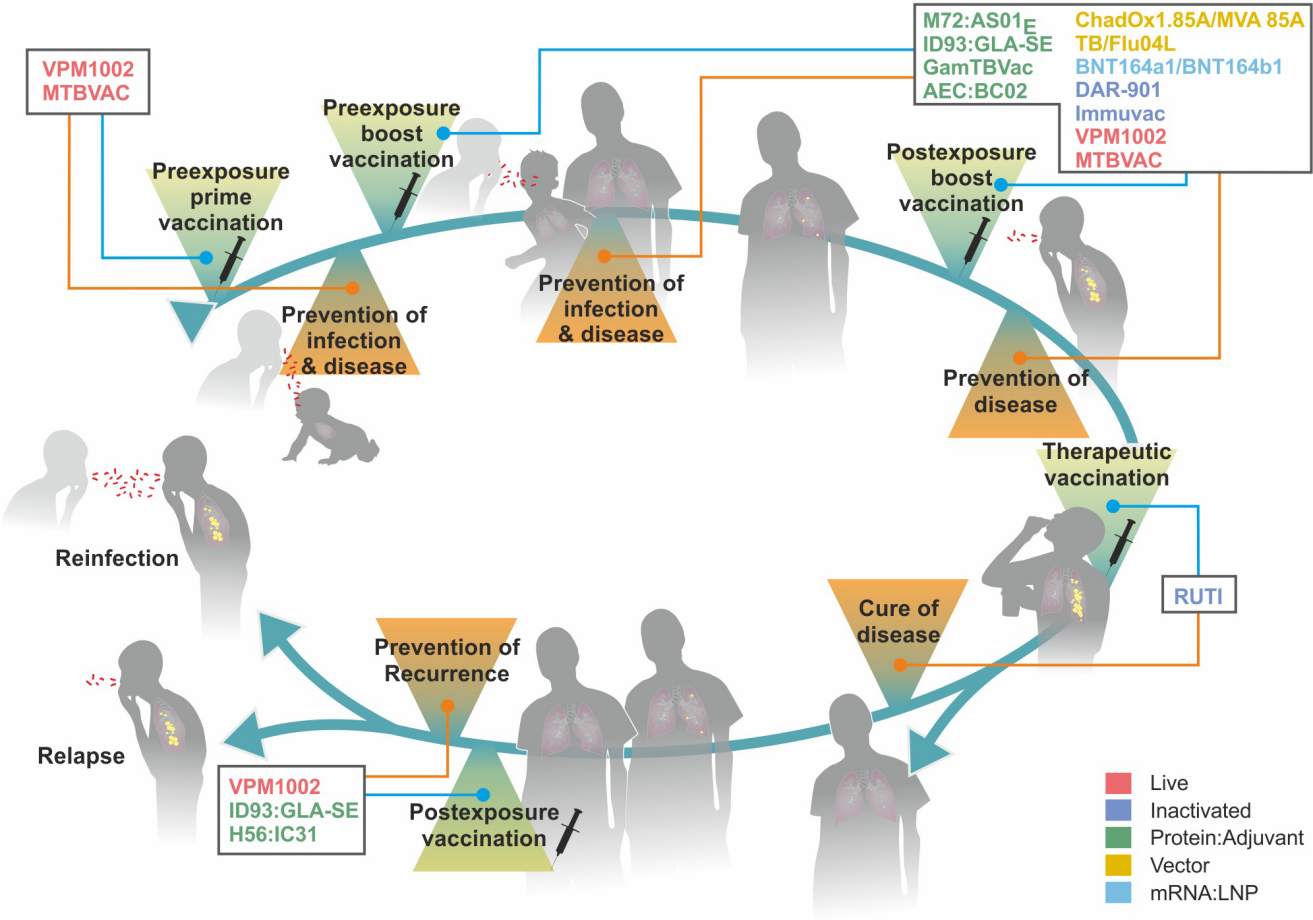
Major stages from infection to active disease in tuberculosis (TB) and target points for different vaccine types. Colors indicate different vaccine platforms. For further details on vaccine types, see **Figure 5**.

**Figure 4** describes the major stages from infection to disease in TB, which serve as targets for intervention by TB vaccines currently under clinical assessment. In the following, different vaccination strategies relevant to TB control, and the value of correlates and surrogates of protection, which are still missing for TB, but availability for Covid-19 will be discussed (119).

### 5.1 Prevention of what?

Principally, vaccines induce a protective immune response against the targeted pathogen with different outcomes that are not mutually exclusive. These are: (i) PoI, (ii) PoD, (iii) prevention of transmission (PoT), and (iv) prevention of recurrence (PoR).

- PoI also leads to PoD and PoT. Infection is best diagnosed by detecting the pathogen or its components. This is feasible as long as the pathogen or its components are present in body sites that are easily accessible, e.g. sputum, urine, or blood. In the case of TB, detection of Mtb or its components generally fails in individuals with LTBI. These individuals are healthy but considered infected with Mtb. Even in patients with active TB, detection of Mtb by sputum microscopy can be missed due to insufficient sensitivity. Diagnosis of LTBI is mostly performed indirectly by measuring the cellular immune response, e.g. by so-called IFN-γ release assays (IGRA) which measure IFN-γ release from white blood cells after antigen-specific stimulation (127–130). Individuals with LTBI have been termed converters. Note that about 10% of close contacts of TB patients do not respond by IGRA and accordingly have been termed nonconverters or resistors (119, 121, 131–133). It is unclear whether nonconverters are true resistors that have cleared or prevented infection or are false nonconverters, which harbor Mtb, but fail to generate an immune response that is measured by IGRA. PoI is mostly accomplished by preventing the pathogen from establishing itself in the host. Frequently, PoI is based on rapid eradication of the pathogen after short-term infection and hence should be more precisely defined as prevention of stable infection. In summary, the precise determination of PoI as a clinical endpoint poses challenges in TB (134). Hence, delineation of the underlying immune mechanisms could provide guidelines for the design of vaccines that target PoI.
- PoD needs to be further subdivided according to the severity of disease, i.e. mild disease, severe disease (hospitalization, intensive care unit), and lethal disease. In the case of Covid-19, vaccines only induce partial PoI but are highly effective in preventing severe disease and lethality. In naïve individuals, PoD is a consequence of PoI. In already infected individuals, PoD can be achieved by pathogen eradication during LTBI or by preventing the pathogen from causing disease, e.g. by its containment in an innocuous stage through maintenance of LTBI. Although Mtb infection is thought to last lifelong, so-called reverters have been described, i.e. individuals who reverted from IGRA^+^ to IGRA^-^ remaining negative over long periods of time (133, 135). The underlying mechanisms remain elusive and false IGRA^-^ due to desensitization cannot be excluded. Given that this reversion reflects the eradication of Mtb, information on the underlying mechanisms could provide helpful guidelines for vaccines aimed at sterilizing PoD.
- PoT is a consequence of PoI and PoD since both directly impact the transmission of Mtb. Although LTBI has long been considered non-contagious, more recent evidence suggests that it can be a major source of transmission. Transmission during LTBI likely occurs during the sub-clinical stage (136–139). Future vaccination strategies need to consider whether vaccines aimed at PoD induce sufficient immune control to prevent Mtb transmission by healthy individuals with sub-clinical TB.
- PoT by itself can serve as a target for future vaccination strategies, notably if vaccine-induced PoD only achieves prevention of severe disease, allowing infection and mild disease.
- PoR targets reinfection or relapse (140). Some individuals who have been cured of TB remain susceptible to reinfection since protective immunity induced by natural Mtb infection is insufficient. In addition, a few Mtb microorganisms may persist even after drug treatment and then cause relapse. PoR is considered a valid target for vaccination. However, it is unlikely that post-TB lung damage is tractable by vaccination.

### 5.2 Preventive and therapeutic vaccination

The major scope of vaccines is to prevent healthy individuals from developing the disease. Yet, therapeutic vaccination in adjunct to chemotherapy is being considered, notably for TB patients suffering from multi- or extensively resistant TB (140). Frequently, vaccines for PoR are grouped as therapeutic vaccines even though recurrence can be caused by reinfection.

### 5.3 Pre- and post-exposure vaccination

By definition, vaccines targeting PoI are administered pre-exposure with the pathogen. Complete PoI also causes PoD and PoT; incomplete PoI may ameliorate disease and transmission. As Covid-19 vaccination campaigns have shown, partial PoI reduces viral load and pathogenicity resulting in efficacious PoD, notably by reducing disease severity. This is likely due to a direct quantitative relationship between viral load and virulence. In the case of TB, such a quantitative relationship is less likely, and partial PoI may delay, but not prevent progression to active TB disease. Theoretically, two options exist that are difficult to differentiate mechanistically. A TB vaccine could either induce PoD or cause containment of Mtb resulting in long-term LTBI. Generally, post-exposure vaccination aims at (i) sterile pathogen eradication before progression to active disease or (ii) long-term maintenance of LTBI. Secondary infection of an individual with LTBI can further complicate the situation.

### 5.4 Prime/boost

Vaccines may need a booster if the prime immunization is insufficient or wanes over time. Even though only little evidence exists, it is often assumed that heterologous prime/boost schemes induce stronger effects, either because the two vaccines cause different immune responses or comprise different antigens. Both effects are considered beneficial if they complement each other. In the case of TB, most vaccine candidates are considered boosters for BCG primed individuals, and only a few as prime vaccines instead of BCG (119).

### 5.5 Surrogates and correlates of protection

#### 5.5.1 Surrogates

A surrogate of protection (SoP) elicited by vaccination is defined as a biologic parameter that in a clinical phase III efficacy trial statistically correlates with vaccine-induced protection (141–143). Typically, SoP is determined by a comparison between the vaccine and the placebo group. SoP, notably if they can be easily determined, facilitates early determination of vaccine effectiveness prior to clinical outcome. Neutralizing antibodies against Spike protein of SARS-CoV-2 are SoP, whereas for TB SoP have not been identified.

#### 5.5.2 Correlates

A correlate of protection (CoP) elicited by vaccination is defined as a biological mechanism, typically an immune mechanism that is induced by immunization and serves as an indicator of protective immunity (142–146). CoP is statistically related to vaccine-induced protection. Comparison of immunized vs. unimmunized (control) individuals will be confounded by the fact that the majority of controls will not develop disease. Given that a vaccine induces protection in some, but not all vaccinees, a comparison of the two immunized groups, i.e. protected vs. unprotected vaccinees provides a strong basis for the definition of a robust CoP. CoP can be used for the definition of surrogate endpoints, i.e. an endpoint that precedes or can be more easily measured than the clinically defined endpoint.

Two groups of CoP need to be distinguished, including direct and indirect CoP. Moreover, CoP induced by infection need not necessarily be identical to CoP induced by vaccination. This is particularly relevant in situations where natural infection does not cause complete protection as in TB. Global gene expression profiling of blood cells and metabolomic analyses of serum led to the design of biosignatures that can potentially predict the progression from LTBI to active TB disease (122–126). Such biosignatures are composed of biomarkers that may or may not be causally linked to the sustenance of LTBI or progression to active TB disease. Biosignature studies on the progression from LTBI to active TB disease as well as on differences between responders and non-responders (see 5.1) can provide important guidelines for the characterization of vaccine-induced CoP. Biosignature studies have provided evidence for a sub-population with sub-clinical TB amongst the LTBI population. Increasing epidemiologic evidence suggests that this healthy sub-population serves as a source of Mtb transmission (136–139).

More recently, biomarkers associated with specific immune responses have been employed for the prediction of TB disease. First, efforts are being made to characterize Mtb-specific antibody profiles comprising Ig isotypes with unique FcR types to identify individuals that progress from LTBI to active TB (91, 93, 147–149). Antibody isotypes related to protection could become promising targets for future vaccines. Second, the identification of Mtb-specific TCR repertoires associated with the outcome of LTBI have been characterized (150). In this study, three groups could be identified based on similarities in TCR sequences: The first group was associated with progression to disease, the second group with maintenance of LTBI, and the third group did not show any association with disease progression or control. Antigenic epitopes related to each group could be identified. PE13, a variable antigen present in both Mtb and BCG as well as CFP10, a cognate of the region of difference (RD)-1 present in Mtb and absent in BCG, were characteristic for infection control. Reciprocally, EspA which is associated with CFP10 was associated with TB progression. Numerous antigens were present in both controllers and progressors, suggesting that they had no direct impact on the course of infection. It should be noted that these analyses focused on CD4 T cells without characterization of their cytokine profile. Future studies extending to functional characterization and other T cell populations, notably CD8 T cells could provide important guidelines for the identification of antigens to be included in, or omitted from, future subunit vaccine candidates. In the long run, an association of functional activities and antigen specificities of B cells and T cells could become important tools for the design of next-generation vaccine candidates.

##### 5.5.2.1 Indirect CoP

Indirect CoP are induced by vaccination either directly and independently from direct CoP, or they are indirect sequelae of direct CoP. They correlate with but are not causally linked to, protection. An example of such indirect CoP is provided by the list of the biomarkers included in biosignatures which potentially predict progression from LTBI to active TB disease (see above).

##### 5.5.2.2 Direct CoP

Direct CoP have also been termed absolute or mechanistic CoP. A direct CoP is not only related to but also responsible for protection. An instructive example of such a causal link is neutralizing antibodies directed against so-called protective antigens, e.g. the Spike protein of SARS-CoV-2 (see also **Figure 2**). An increase in neutralizing antibody titers against the RBD of the Spike protein is not only directly related to the strength of vaccine-induced immunity, but can also be harnessed for measuring vaccine efficacy. Advantageously, neutralizing antibodies express both relevant functions (blocking of viral entry into host cells) and relevant specificity (targeting the RBD of the Spike protein). Accordingly, the omission of the Spike protein from a vaccine against Covid-19 will fail to produce a protective immune response mediated by neutralizing antibodies. Neutralizing antibodies that prevent Mtb from infection do not exist because Mtb is engulfed by mononuclear phagocytes through a variety of active uptake mechanisms involving numerous receptors. Antigen-specific CD4 T cells are directly involved in protective immunity against TB. Yet, their biological functions depend on mediators such as cytokines and chemokines that act on other immune cells, e.g. stimulation of CD8 T cells to express cytolytic activity, B cells to secrete antibodies or mononuclear phagocytes to express bacteriostatic or bactericidal activity. Moreover, antigen specificity of T cells need not be directly linked to T cell function. In TB immunity that sustains LTBI and therefore prevents active TB disease may differ from immunity that prevents infection or causes sterile eradication of Mtb.

#### 5.5.3 Relevance to vaccine development

For discussion here, it is clear that rational vaccine development will enormously benefit from the identification of direct CoP (151). The most straightforward CoP are antigen-specific antibodies with neutralizing activity, which cause PoI by preventing pathogen entry into host cells. This activity becomes more complex in situations, in which antibodies contribute to protection via FcR-mediated effector functions of antibodies, such as complement activation, opsonization, or ADCC. The most complex situation arises when infection is primarily controlled by cell-mediated immunity. First, the measurement of antigen specificity of T cells is more challenging, and second, the function of T cells is ultimately mediated by effector molecules or effector cells. CD8 T cells, which contribute to protection as CTL are relatively straightforward because they act directly via their ‘own’ effector molecules (e.g. granzyme, granulysin, perforin). The most complex situation arises for CD4 T cells, which primarily act through soluble mediators such as chemokines and cytokines to stimulate other cells to perform effector mechanisms including mononuclear phagocytes, granulocytes, B cells, and CD8 T cells.

## 6 TB vaccine candidates

### 6.1 BCG: from early testing to most recent trials

BCG is one of the most widely used vaccines globally only exceeded by Covid-19 vaccines. Some four billion doses have been rolled out since its first human use in the 1920s (36, 152). The term BCG stems from the two developers, Albert Calmette and Camille Guérin, who succeeded in attenuating *M. bovis*, the causative agent of bovine TB by passaging it > 230 times *in vitro* using ox bile to promote attenuation (153). This vaccine has been designed for the prevention of TB in neonates with a high risk of progression to severe extrapulmonary disease including miliary TB due to dissemination of Mtb to diverse organs. BCG accomplishes this goal, at least in part, but fails to protect against pulmonary TB, notably in adolescents and adults (154). The vaccine was originally administered in three doses given orally and this regimen was later changed to intradermal administration of a single dose. In some countries, revaccination with BCG has been performed, notably in neonates and infants lacking signs of vaccine take and sometimes also in adolescents and adults. Generally, however, BCG boosters are not recommended because of the potential risk of adverse events.

**Figure 5:**
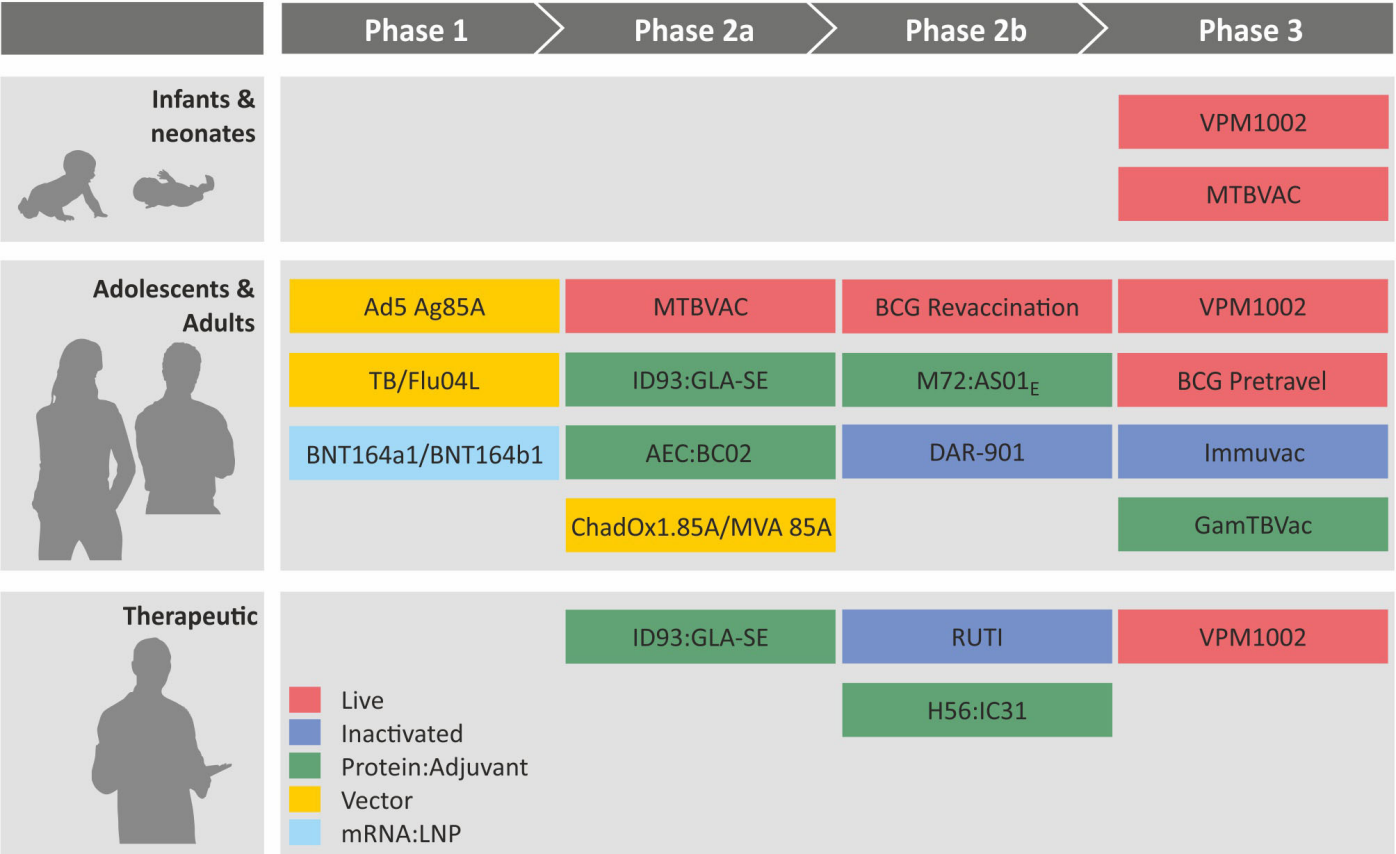
Clinical pipeline of major tuberculosis vaccines from Phase I to Phase III. Colors indicate different vaccine platforms. For further details, see article discussion.

A recent study assessed BCG revaccination of adolescents and adults without signs of Mtb infection (see **Figure 5**). This study (NCT02075203) found ca. 45% protection against stable Mtb infection indicated by IGRA (155). It is noteworthy that stable, but not transient, infection was prevented by BCG revaccination. It has been argued, therefore, that in the BCG immunized group, Mtb was able to establish itself for a short time period, but was subsequently eliminated by mononuclear phagocytes expressing trained immunity (see 4) (62). Consistently, BCG revaccination caused significant protection against upper respiratory viral infections over controls (note that lower respiratory infections were not observed in either group). A larger confirmatory trial with BCG is underway (NCT04152161). Another study with BCG, which is currently in phase III, assesses the value of BCG for pre-travel vaccination (see **Figure 5**). The clinical endpoint is PoI in healthy adult travelers from low incidence countries who are at risk of exposure to Mtb in high burden countries (NCT04453293). Completion of this study is expected in 2025.

### 6.2 Experimental BCG studies as the first step towards CoP

A breakthrough study in which non-human primates (NHP) had been immunized with BCG by intravenous administration resulted in the sterile eradication of Mtb in the majority of animals. Whilst this way of administration is hampered by the risk of adverse events, it provides proof of concept that sterilizing immunity can be induced even though the underlying mechanisms have to be fully revealed (156). This model was harnessed for the identification of immune mechanisms and biosignatures relevant to protective immunity. By using a lower dose of BCG given intravenously, sterile protection could be induced in about half of the experimental animals. Comparison of protected and unprotected NHP revealed that an abundance of polyfunctional T cells that co-express TNF together with IFN-γ or with IL-17 as well as an abundance of NK cells correlated with protection two months after immunization and before challenge with Mtb (157). In parallel, blood transcriptional correlates were determined. Biosignatures determined two days after immunization correlated with the pulmonary immune responses measured after one to two months (see above) and could predict protection against Mtb challenge after six months (158). These modules included type I IFN as well as Rag-I-like signaling pathways. These studies in NHP both on the cellular and transcriptional level provide the first evidence that vaccine-induced CoP can be defined. As a caveat, it should be noted, however, that the transcriptional module can be affected by viral infections occurring during vaccine trials which induce similar biosignatures. Furthermore, in adolescents and adults, a high proportion of individuals already have LTBI demanding post-exposure vaccination with Mtb which may differ from the pre-exposure situation studied in NHP. Finally, it needs to be clarified whether the gene expression profiles represent direct or indirect CoP. Direct CoP could be harnessed for further refinement of novel vaccine candidates.

### 6.3 Vaccine candidates in clinical trials

Currently, more than a dozen vaccine candidates against TB are progressing through the clinical trial pipeline and have advanced to different stages, phase I, phase II, or phase III (**Figure 5**). These include five protein adjuvant vaccines and three viral vectored vaccines as cognates of subunit vaccine candidates as well as three inactivated and two attenuated vaccines as members of whole cell vaccine candidates. Most recently mRNA : LNP vaccines have entered phase I safety assessment as the latest addition to the group of subunit vaccine candidates.

#### 6.3.1 Subunit vaccines

##### 6.3.1.1 Protein:adjuvants

- **H56:IC31** is a fusion protein of three antigens (ESAT-6, a prominent Mtb antigen in the RD-1 region + Ag85B, a member of the Ag85 family of mycolyl-transferases + Rv2660c, a dormancy antigen) in the IC31 adjuvant (cationic peptide + TLR9 agonist) (159). It has successfully completed several phase I trials for safety and immunogenicity and is currently being tested in a clinical phase II trial (NCT03512249) for therapeutic purposes (PoR).
- **ID93:GLA-SE** is based on a fusion protein of four antigens (Rv2608, a PPE family member + Rv3619, a virulence factor + Rv3620, another virulence factor + Rv1813, a dormancy antigen) in the GLA-SE adjuvant (an oil-in-water emulsion + TLR4 agonist) (160). It is considered for preventive and therapeutic purposes (PoR) and has successfully completed a phase IIa trial (NCT03806686).
- **AEC : BC02** comprises three antigens (Ag85B, a member of the Ag85 family of mycolyl-transferases + ESAT-6 + CFP10, two important antigens in the RD-1 region) in the BC02 adjuvant (TLR-9 agonist CpG in alum) (28). It has reached a phase II clinical trial (NCT05284812).
- **GamTBvac** is composed of ESAT-6 + CFP10 (two important antigens in the RD-1 region) + Ag85A (a member of the Ag85 family of mycolyl-transferases) with a modified Dextran-binding domain formulated with the TLR-9 agonist CpG as adjuvant (161). This vaccine candidate has entered a phase III trial for the prevention of TB in adolescents and adults (NCT04975737). Completion is expected in 2025.
- **M72:AS01_E_** comprises a fusion protein of two antigens (Rv1196, a PPE family member + Rv0125, a peptidase) in the AS01_E_ (liposome + TLR4 agonist). The M72:AS01_E_ showed ca. 54% protection against progression to active TB from LTBI in a phase IIb prevention trial (162, 163). In this trial (NCT01755598), M72:AS01_E_ was given as a post-exposure boost vaccine in adults and adolescents with LTBI who had been BCG primed as infants. This vaccine is planned for a larger phase II/III trial to validate its protective efficacy in PLWH (NCT04556981).
- Two similar mRNA : LNP vaccines against TB have entered the clinical trial pipeline. BNT164a1 and BNT164b1 encoding multiple Mtb antigens of undisclosed identity are in phase I trials in BCG-vaccinated HIV-negative individuals (NCT 05547464) and in IGRA-negative, BCG naïve individuals (NCT 05537038). Thus, the two mRNA : LNP vaccine candidates are considered both as a prime vaccine in Mtb-uninfected and BCG-unvaccinated individuals and as a boost vaccine in BCG-immunized, Mtb-infected (LTBI) and naïve individuals.

##### 6.3.1.2 Viral vectors

- **Ad5Ag85A** is based on a nonreplicating Ad vector expressing Ag85A (a member of the Ag85 mycolyl-transferase family) (164). It has completed a phase I trial for safety and immunogenicity after aerosol inhalation (NCT02337270). Work with this vaccine candidate has been discontinued.
- **TB/Flu04L** comprises a non-replicating influenza virus as a vector expressing Ag85A (a member of the Ag85 mycolyl-transferase family) and ESAT-6 (a prominent Mtb antigen in the RD-1 region) (31). It has successfully completed a phase I clinical trial for safety after intranasal and sublingual administration (NCT03017378).
- **ChAdOx1.85A/MVA** is given in a heterologous prime/boost scheme, where ChAdOx serves as the prime and MVA as a boost. Both vectors express Ag85 and thus differ in the vector, not in the antigen (165). This heterologous vaccination regimen has recently entered a phase IIa trial (NCT03681860). The ChAdOx1.85A vaccine has also completed a comparative phase I trial for aerosol versus intramuscular vaccination (NCT04121494). Previously, a completed phase IIb trial with MVA85A alone had failed to provide evidence for protective efficacy (NCT00480558) (166–168).

#### 6.3.2 Whole cell vaccines

##### 6.3.2.1 Inactivated

- **RUTI** which is exclusively targeted for the therapy of TB, notably multidrug-resistant (MDR) or extensively drug-resistant (XDR)-TB in adjunct to chemotherapy has reached phase IIb stage (NCT04919239). RUTI is a killed and detoxified Mtb preparation in liposome suspension (169).
- **DAR-901** has been tested for the prevention of TB in adolescents and adults. A phase IIb trial has been completed without evidence for protective efficacy (NCT02712424). This vaccine comprises a killed *M. obuense* preparation (170).
- **Immuvac**, the most advanced inactivated vaccine is based on killed M*. indicus pranii* (171). This vaccine provided some evidence for therapeutic protection when given in adjunct to chemotherapy (NCT00265226). It is currently tested head-to-head with VPM1002 (CTRI/2019/01/017026) in a phase III trial for PoD with estimated completion in 2024.

##### 6.3.2.2 Attenuated

- **MTBVAC** is a viable vaccine candidate which is tested for TB prevention in infants and adolescents/adults. It has reached a phase III trial in infants (NCT04975178), and completion is expected in 2029. It is a live Mtb vaccine candidate that had been attenuated by genetic deletion of two independent loci that regulate more than 100 genes in Mtb (172).
- **VPM1002** is an improved BCG vaccine candidate, in which the urease C gene has been replaced by the listeriolysin gene (152, 173). It is currently undergoing three phase 3 trials: (i) PoI and PoD in neonates in comparison to BCG (NCT04351685) with expected completion by 2025; (ii) PoD in adolescent and adult household contacts of recently diagnosed TB patients head-to-head with Immuvac (CTRI/2019/01/017026) with estimated completion in 2024; (iii) PoR in individuals who had completed TB chemotherapy (NCT03152903) with estimated completion in 2024.

### 6.4 Concluding remarks on TB vaccine candidates

Globally an estimated 1.7 billion individuals live with LTBI, of whom approximately 10% develop active disease, the majority within the first year, but others after decades. Therefore, vaccines need to be considered for pre- and post-Mtb exposure and for induction of long-lasting protection not only in individuals who develop active TB within less than 12 months but also in those who become ill much later.

As outlined in the above review, the genome of Mtb comprises some 4000 protein-encoding genes, which in principle could all be target antigens for vaccine-induced immunity (174). Several of these antigens are regulated, with some proteins being more abundant during active TB disease and others during LTBI. Post-exposure vaccination of individuals with LTBI carrying dormant Mtb, therefore, may depend on antigens different from those in a vaccine that prevents infection with active Mtb.

In contrast to SARS-CoV-2, where neutralizing antibodies are of critical importance, evidence is missing as to whether neutralizing antibodies are generated in TB. Accordingly, protective antigens are absent. Increasing evidence suggests a role for non-neutralizing antibodies in protective immunity which activate different effector functions. Moreover, T cells are essential for protection and pathology and strong evidence exists that a fine-tuned balance between innate and acquired immune cells is critical for protective immunity. Based on these features neither surrogates nor direct correlates of protection against TB have been identified thus far.

## 7 Lessons from Covid-19 for TB vaccine R&D

The devastating health crisis created by Covid-19 provided pivotal lessons for future epidemic, endemic, and pandemic control measures at all levels including vaccine R&D for TB. Lessons of general relevance include the need for stronger healthcare systems, improved infection control measures, better preparedness and resilience to emerging and existing health threats, and better public health education.

More specific guidelines from the Covid-19 crisis that are relevant for TB vaccine R&D include, financial support for TB vaccine R&D is of critical importance. At present, support is still insufficient despite a slight increase over the last decade, costing approximately $1 billion US dollars per year (175). The immediate support by public, philanthropic, and private partners for Covid-19 vaccine R&D up to $100 billion US dollars (14). This unprecedented funding demonstrates the impact that early financial investment can have on vaccine R&D for health. It has to be voiced more clearly that in the long run, investment in TB vaccine R&D will pay back (176). The current financial burden of TB has been estimated in the order of $100 billion US dollars annually. Hence, public-philanthropic-private partnerships should be formed if the industry hesitates to invest in TB vaccines because of assumed low profit. A recent example of a philanthropic-private partnership is the handing over of the TB vaccine, M72:AS01_E,_ for phase II/III clinical trial testing from GlaxoSmithKline to the Bill and Melinda Gates Foundation and the Wellcome Trust (177).

The global response to the Covid-19 crisis fostered a stronger collaborative spirit among researchers from both public and private entities, which profoundly accelerated vaccine development. This lesson should be applied to improve joint research and resource mobilization for TB vaccine R&D. A recent example is the head-to-head phase III clinical trial performed by the Indian Council of Medical Research to compare protection against TB by the attenuated vaccine, VPM1002, and the inactivated vaccine, Immuvac (178). In a similar vein, late stage vaccine trials should not only aim to provide information on the vaccine candidate under trial but also to generate information for the informed design of next-generation vaccine candidates.

The transition from preclinical to clinical studies has frequently been termed the ‘valley of death’ due to the many obstacles that can occur. During the Covid-19 crisis, the regulatory processes for vaccines were markedly expedited by streamlined regulatory processes to mitigate such obstacles, at least partially. TB vaccine R&D could similarly benefit from streamlined regulatory processes without any curtailment in safety and efficacy standards (20). Related to this, adaptive clinical trial design can further contribute to accelerated clinical vaccine testing (15, 134). Both strategies can speed up the clinical development pipeline without compromising safety and efficacy standards.

Once a better efficacy and/or safety profile for a novel TB vaccine over BCG has been established, vaccine manufacturing capacity will become a critical factor (179). Hence, appropriate manufacturing capacities need to be established early on: at the latest, in parallel to a phase III vaccine trial. Since this is best accomplished by facilities with high manufacturing capacity meeting global demands, appropriate partnerships need to be established and investment into expanded manufacturing capabilities needs to be mobilized. The Covid-19 pandemic provides lessons, some of which should be followed and others modified or avoided. A positive example is the agreement between the startup company BioNTech and the big pharma company Pfizer to develop, test, and deploy Comirnaty as fast as possible. On the other hand, the COVAX enterprise ultimately failed to achieve equitable vaccine distribution across the globe (180, 181).

Another important aspect of this topic is equitable access to TB vaccines at low cost, which needs to be guaranteed for low- and middle-income countries, not the least because they face the highest TB burden (17, 182). One step towards this could be the establishment of vaccine manufacturing capacities in regions where TB vaccines are needed most. This strategy includes not only manufacturing capacities but also strong educational and training activities to ensure successful TB vaccine production and deployment from local manufacturers (21). The largest vaccine manufacturer by dose is the Serum Institute of India Pvt. Ltd., which is based in India, a country with a high prevalence of TB. For regions without manufacturing capacities, the founding of WHO mRNA vaccine hubs on the African continent provides precedent from the Covid-19 field for this strategy (183).

Covid-19 vaccination campaigns have highlighted the importance of robust surveillance and monitoring systems that track the effectiveness and adverse events of the newly deployed vaccines. These lessons need to be adopted in modified form for TB from rollout to long-term surveillance of vaccines, notably since long-term protection is essential for TB control.

The impact of Covid-19 vaccination programs has saved millions of lives. Yet, in a small proportion of the global population, in both the North and the South, vaccine hesitancy and even aggressive vaccine opposition arose (184). Many diverse reasons account for vaccine hesitancy and denial including distrust of traditional political authorities (185). Hence, multidimensional approaches will be required to mitigate these challenges (186, 187). Successful TB vaccine rollout needs to be accompanied by a build-up in public trust through engaging and educating communities that suffer from high TB burden and addressing their concerns.

A major game changer emerged during the Covid-19 vaccine crisis, namely the creation of viral-vectored and mRNA : LNP vaccines as novel vaccine platforms. Whilst vector-based vaccines have already been included in the TB vaccine R&D portfolio, mRNA : LNP vaccines represent a novelty. This versatile platform must be included in the TB vaccine R&D pipeline. A major vaccine developer, BioNTech, already started a phase I safety trial for mRNA : LNP vaccines that encode various TB antigens. Assuming that subunit vaccines covering a small number of antigens can target Mtb with sufficient efficiency to provide long-term protection it is likely that mRNA : LNP vaccines can become major players for TB control.

## 8 Conclusion

TB has been around for centuries, claiming more than a billion lives (9). Despite its threat, the TB crisis has remained largely silent. Indeed, TB morbidity and mortality have been on the decline over recent decades; yet this decline is far too meager and alone it will not enable us to reach the goal of ending TB by 2030 as proposed by the Stop TB Partnership and the WHO (188, 189). This decline even reversed with the emergence of SARS-CoV-2 with an estimated 10-11 million people acquiring active TB and 1.6 million people dying. In 2018, a High-Level Meeting of the United Nations (UN) General Assembly made a strong commitment to end TB by 2030 (190–192). To achieve the goal, the UN is committed to creating “an environment conducive to research and development for new tools for TB”. Accordingly, the commitment was made “to mobilize sufficient and sustainable financing with the aim of increasing overall global investments to 2 billion US dollars [ … ] in funding annually for tuberculosis research” (191). This noble goal was interrupted by Covid-19. Hence, in 2023, a second High-Level Meeting on TB will be convened by the UN (193).

A strong commitment to ending TB is urgently needed. Otherwise, the WHO goal of reducing TB incidence by 50% and the numbers of TB deaths by 75% between 2015 and 2025 will be missed, notably because by 2021 only 10% reduction in TB incidence and 5.9% reduction in TB deaths had been accomplished. In 2023, 30,000 people develop active TB every day and approximately 4,200 of them will die of this disease. By 2050, four million deaths will occur leading to an economic loss of $13 billion US Dollars (192). Better intervention measures are urgently needed and TB vaccines play a major role in this endeavor.

As has been discussed in this review, TB vaccine R&D cannot replicate the success story of Covid-19 vaccine R&D. Yet, by building on the experience gathered during the Covid-19 pandemic, the conditions for TB can be changed for the better. In the aftermath of the Covid-19 crisis, a working group had been established under the leadership of E.J. Sirleaf, former President of Liberia, and H. Clark, former Prime Minister of New Zealand, on ‘How an Outbreak Became a Pandemic’ under the ethos, ‘Covid-19: Make it the Last Pandemic’ (7, 8). Their concluding comment stated that their “message for change is clear: no more pandemic. If we fail to take this goal seriously, we will condemn the world to successive catastrophes”. They go on to outline how the demands of this task are “large and challenging, but the price is even larger and more rewarding. With so many lives at stake, now is the time to resolve ”. This call to prevent the next pandemic can be rephrased as task for future control of the ongoing TB pandemic: the “message for change is clear: No more TB. If we fail to take this goal seriously, we will condemn the world to continued catastrophes. The ask is large and challenging, but the price is even larger and more rewarding. With so many lives at stake, now is the time to resolve”.

## Author contributions

SK: Conceptualization, Writing – original draft.

## References

[1] World Health Organization. COVID-19 dashboard (2020). Available at: https://covid19.who.int/.

[2] Mathieu E Ritchie H Rodés-Guirao L Appel C Giattino C Hasell J. Coronavirus pandemic (COVID-19). Our World Data (2020).

[3] Msemburi W Karlinsky A Knutson V Aleshin-Guendel S Chatterji S Wakefield J. The WHO estimates of excess mortality associated with the COVID-19 pandemic. Nature (2022) 613:130–7. doi: 10.1038/s41586-022-05522-2 PMC9812776 36517599

[4] Hogan AB Jewell BL Sherrard-Smith E Vesga JF Watson OJ Whittaker C. Potential impact of the COVID-19 pandemic on HIV, tuberculosis, and malaria in low-income and middle-income countries: a modelling study. Lancet Glob Health (2020) 8:e1132–41. doi: 10.1016/S2214-109X(20)30288-6 PMC7357988 32673577

[5] World Health Organization. Global tuberculosis report (2022). Available at: https://www.who.int/teams/global-tuberculosis-programme/tb-reports/global-tuberculosis-report-2022.

[6] World Health Organization. Coronavirus disease (COVID-19) pandemic. Available at: https://www.who.int/emergencies/diseases/novel-coronavirus-2019.

[7] How an outbreak became a pandemic: The defining moments of the COVID-19 pandemic. The Independent Panel for Pandemic Preparedness and Response (2021). Available at: https://theindependentpanel.org/wp-content/uploads/2021/05/How-an-outbreak-became-a-pandemic_final.pdf.

[8] COVID-19: Make it the Last Pandemic. The Independent Panel for Pandemic Preparedness and Response (2021). Available at: https://theindependentpanel.org/wp-content/uploads/2021/05/COVID-19-Make-it-the-Last-Pandemic_final.pdf.

[9] Paulson T. Epidemiology: A mortal foe. Nature (2013) 502:S2–3. doi: 10.1038/502S2a 24108078

[10] Ivanova O Hoffmann VS Lange C Hoelscher M Rachow A. Post-tuberculosis lung impairment: systematic review and meta-analysis of spirometry data from 14 621 people. Eur Respir Rev (2023) 32:220221. doi: 10.1183/16000617.0221-2022 37076175 PMC10113954

[11] Nightingale R Carlin F Meghji J McMullen K Evans D van der Zalm MM. Post-TB health and wellbeing. Int J Tuberc Lung Dis (2023) 27:248–83. doi: 10.5588/ijtld.22.0514 PMC10094053 37035971

[12] Ayouba A Thaurignac G Morquin D Tuaillon E Raulino R Nkuba A. Multiplex detection and dynamics of IgG antibodies to SARS-CoV2 and the highly pathogenic human coronaviruses SARS-CoV and MERS-CoV. J Clin Virol (2020) 129:104521. doi: 10.1016/j.jcv.2020.104521 32623350 PMC7308014

[13] Hu B Guo H Zhou P Shi Z-L. Characteristics of SARS-coV-2 and COVID-19. Nat Rev Microbiol (2021) 19:141–54. doi: 10.1038/s41579-020-00459-7 PMC7537588 33024307

[14] Florio M Gamba S Pancotti C. Mapping of long-term public and private investments in the development of Covid-19 vaccines. Eur Parliament COVI Committee (2023) 1–95. doi: 10.2861/481388

[15] Corey L Miner MD. Accelerating clinical trial development in vaccinology: COVID-19 and beyond. Curr Opin Immunol (2022) 76:102206. doi: 10.1016/j.coi.2022.102206 35569415 PMC9020485

[16] Edwards DK Carfi A. Messenger ribonucleic acid vaccines against infectious diseases: current concepts and future prospects. Curr Opin Immunol (2022) 77:102214. doi: 10.1016/j.coi.2022.102214 35671599 PMC9612403

[17] Alakija A. Leveraging lessons from the COVID-19 pandemic to strengthen low-income and middle-income country preparedness for future global health threats. Lancet Infect Dis (2023) 23(8):e310-e317. doi: 10.1016/S1473-3099(23)00279-7 37290474

[18] Agarwal R Reed T. Finance vaccine equity: funding for day-zero of the next pandemic IMF working paper no. 2022/099 (2022). Available at: https://ssrn.com/abstract=4129564.

[19] Sah P Vilches TN Moghadas SM Pandey A Gondi S Schneider EC. Return on investment of the COVID-19 vaccination campaign in new york city. JAMA Netw Open (2022) 5:e2243127. doi: 10.1001/jamanetworkopen.2022.43127 36409495 PMC9679875

[20] Scheppler L De Clercq N McGoldrick M Dias J. Regulatory Harmonization and Streamlining of Clinical Trial Applications globally should lead to faster clinical development and earlier access to life-saving vaccines. Vaccine (2021) 39:790–6. doi: 10.1016/j.vaccine.2020.11.077 33422378

[21] Kana BD Arbuthnot P Botwe BK Choonara YE Hassan F Louzir H. Opportunities and challenges of leveraging COVID-19 vaccine innovation and technologies for developing sustainable vaccine manufacturing capabilities in Africa. Lancet Infect Dis (2023) 23:e288–300. doi: 10.1016/S1473-3099(22)00878-7 37290473

[22] Pollard AJ Bijker EM. A guide to vaccinology: from basic principles to new developments. Nat Rev Immunol (2021) 21:83–100. doi: 10.1038/s41577-020-00479-7 33353987 PMC7754704

[23] Chauhan N Tiwari S Iype T Jain U. An overview of adjuvants utilized in prophylactic vaccine formulation as immunomodulators. Expert Rev Vaccines (2017) 16:491–502. doi: 10.1080/14760584.2017.1306440 28285554

[24] Didierlaurent AM Laupèze B Di Pasquale A Hergli N Collignon C Garçon N. Adjuvant system AS01: helping to overcome the challenges of modern vaccines. Expert Rev Vaccines (2017) 16:55–63. doi: 10.1080/14760584.2016.1213632 27448771

[25] Stertman L Palm A-KE Zarnegar B Carow B Lunderius Andersson C Magnusson SE. The Matrix-M™ adjuvant: A critical component of vaccines for the 21st century. Hum Vaccin Immunother (2023) 19:2189885. doi: 10.1080/21645515.2023.2189885 37113023 PMC10158541

[26] Turley JL Lavelle EC. Resolving adjuvant mode of action to enhance vaccine efficacy. Curr Opin Immunol (2022) 77:102229. doi: 10.1016/j.coi.2022.102229 35779364

[27] Hu Z Lu S-H Lowrie DB Fan X-Y. Research advances for virus-vectored tuberculosis vaccines and latest findings on tuberculosis vaccine development. Front Immunol (2022) 13:895020. doi: 10.3389/fimmu.2022.895020 35812383 PMC9259874

[28] Lu J Guo X Wang C Du W Shen X Su C. Therapeutic effect of subunit vaccine AEC/BC02 on mycobacterium tuberculosis post-chemotherapy relapse using a latent infection murine model. Vaccines (2022) 10:825. doi: 10.3390/vaccines10050825 35632581 PMC9145927

[29] McCann N O'Connor D Lambe T Pollard AJ. Viral vector vaccines. Curr Opin Immunol (2022) 77:102210. doi: 10.1016/j.coi.2022.102210 35643023 PMC9612401

[30] Jacob-Dolan C Barouch DH. COVID-19 vaccines: adenoviral vectors. Ann Rev Med (2022) 73:41–54. doi: 10.1146/annurev-med-012621-102252 34609905 PMC8795482

[31] Buzitskaya Z Stosman K Khairullin B Kassenov M Nurpeisova A Abylai Sansyzbay A. A new intranasal influenza vector-based vaccine TB/FLU-04L against tuberculosis: preclinical safety studies. Drug Res (2022) 72:255–8. doi: 10.1055/a-1785-3936 35318622

[32] Kieh M Richert L Beavogui AH Grund B Leigh B D'Ortenzio E. Randomized trial of vaccines for zaire ebola virus disease. N Engl J Med (2022) 387:2411–24. doi: 10.1056/NEJMoa2200072 36516078

[33] Hald Albertsen C Kulkarni JA Witzigmann D Lind M Petersson K Simonsen JB. The role of lipid components in lipid nanoparticles for vaccines and gene therapy. Adv Drug Deliv Rev (2022) 188:114416. doi: 10.1016/j.addr.2022.114416 35787388 PMC9250827

[34] Hogan MJ Pardi N. mRNA vaccines in the COVID-19 pandemic and beyond. Annu Rev Med (2022) 73:17–39. doi: 10.1146/annurev-med-042420-112725 34669432

[35] Verbeke R Hogan MJ Loré K Pardi N. Innate immune mechanisms of mRNA vaccines. Immunity (2022) 55:1993–2005. doi: 10.1016/j.immuni.2022.10.014 36351374 PMC9641982

[36] Lange C Aaby P Behr MA Donald PR Kaufmann SHE Netea MG. 100 years of Mycobacterium bovis bacille Calmette-Guérin. Lancet Infect Dis (2022) 22:e2–e12. doi: 10.1016/S1473-3099(21)00403-5 34506734 PMC11967564

[37] World Health Organization. Emergency use listing (EUL). Available at: https://www.who.int/teams/regulation-prequalification/eul.

[38] Bao Y He L Miao B Zhong Z Lu G Bai Y. BBIBP-CorV vaccination accelerates anti-viral antibody responses in heterologous Omicron infection: A retrospective observation study in Shanghai. Vaccine (2023) 41:3258–65. doi: 10.1016/j.vaccine.2023.03.070 37085449

[39] Ralise AEG Camargo TM Marson FAL. Phase 4 clinical trials in the era of the Coronavirus Disease (COVID-19) pandemic and their importance to optimize the COVID-19 vaccination. Hum Vaccin Immunother (2023) 19:2234784. doi: 10.1080/21645515.2023.2234784 37449956 PMC10351445

[40] Hotez PJ Bottazzi ME. Whole inactivated virus and protein-based COVID-19 vaccines. Ann Rev Med (2022) 73:55–64. doi: 10.1146/annurev-med-042420-113212 34637324

[41] Taucher C Lazarus R Dellago H Maurer G Weisova P Corbic-Ramljak I. Safety and immunogenicity against ancestral, Delta and Omicron virus variants following a booster dose of an inactivated whole-virus COVID-19 vaccine (VLA2001): Interim analysis of an open-label extension of the randomized, controlled, phase 3 COV-COMPARE trial. J Infect (2023) 87(3):242–54. doi: 10.1016/j.jinf.2023.06.022 37406777

[42] Rose W Raju R Babji S George A Madhavan R Leander Xavier JV. Immunogenicity and safety of homologous and heterologous booster vaccination of ChAdOx1 nCoV-19 (COVISHIELD™) and BBV152 (COVAXIN®): a non-inferiority phase 4, participant and observer-blinded, randomised study. Lancet Reg Health Southeast Asia (2023) 12:100141. doi: 10.1016/j.lansea.2023.100141 36712811 PMC9870748

[43] Madhi SA Kwatra G Richardson SI Koen AL Baillie V Cutland CL. Durability of ChAdOx1 nCoV-19 (AZD1222) vaccine and hybrid humoral immunity against variants including omicron BA.1 and BA.4 6 months after vaccination (COV005): a *post-hoc* analysis of a randomised, phase 1b–2a trial. Lancet Infect Dis (2023) 23:295–306. doi: 10.1016/S1473-3099(22)00596-5 36273491 PMC9584570

[44] Ferrara F Mancaniello C Varriale A Sorrentino S Zovi A Nava E. COVID-19 mRNA vaccines: A retrospective observational pharmacovigilance study. Clin Drug Investig (2022) 42:1065–74. doi: 10.1007/s40261-022-01216-9 PMC9589581 36274082

[45] Jin P Guo X Chen W Ma S Pan H Dai L. Safety and immunogenicity of heterologous boost immunization with an adenovirus type-5-vectored and protein-subunit-based COVID-19 vaccine (Convidecia/ZF2001): A randomized, observer-blinded, placebo-controlled trial. PloS Med (2022) 19:e1003953. doi: 10.1371/journal.pmed.1003953 35617368 PMC9187065

[46] Underwood E Dunkle LM Madhi SA Gay CL Heath PT Kotloff KL. Safety, efficacy, and immunogenicity of the NVX-CoV2373 vaccine. Expert Rev Vaccines (2023) 22:501–17. doi: 10.1080/14760584.2023.2218913 37246757

[47] Ku JH Sy LS Qian L Ackerson BK Luo Y Tubert JE. Effectiveness of a fourth dose of mRNA-1273 against COVID-19 among older adults in the United States: Interim results from an observational cohort study. Vaccine (2023) 41:4212–9. doi: 10.1016/j.vaccine.2023.06.016 PMC10239903 37301708

[48] Moreira ED Kitchin N Xu X Dychter SS Lockhart S Gurtman A. Safety and efficacy of a third dose of BNT162b2 covid-19 vaccine. N Engl J Med (2022) 386:1910–21. doi: 10.1056/NEJMoa2200674 PMC9006787 35320659

[49] Pérez-Then E Lucas C Monteiro VS Miric M Brache V Cochon L. Neutralizing antibodies against the SARS-CoV-2 Delta and Omicron variants following heterologous CoronaVac plus BNT162b2 booster vaccination. Nat Med (2022) 28:481–5. doi: 10.1038/s41591-022-01705-6 PMC8938264 35051990

[50] Murray SM Ansari AM Frater J Klenerman P Dunachie S Barnes E. The impact of pre-existing cross-reactive immunity on SARS-CoV-2 infection and vaccine responses. Nat Rev Immunol (2022) 23:304–16. doi: 10.1038/s41577-022-00809-x PMC9765363 36539527

[51] Jacobs JL Haidar G Mellors JW. COVID-19: challenges of viral variants. Annu Rev Med (2023) 74:31–53. doi: 10.1146/annurev-med-042921-020956 35850493

[52] Chen Y Zhao X Zhou H Zhu H Jiang S Wang P. Broadly neutralizing antibodies to SARS-CoV-2 and other human coronaviruses. Nat Rev Immunol (2023) 23:189–99. doi: 10.1038/s41577-022-00784-3 PMC9514166 36168054

[53] Sette A Sidney J Crotty S. T cell responses to SARS-coV-2. Ann Rev Immunol (2023) 41:343–73. doi: 10.1146/annurev-immunol-101721-061120 36750314

[54] Wherry EJ Barouch DH. T cell immunity to COVID-19 vaccines. Science (2022) 377:821–2. doi: 10.1126/science.add2897 35981045

[55] Netea MG Ziogas A Benn CS Giamarellos-Bourboulis EJ Joosten LAB Arditi M. The role of trained immunity in COVID-19: Lessons for the next pandemic. Cell Host Microbe (2023) 31:890–901. doi: 10.1016/j.chom.2023.05.004 37321172 PMC10265767

[56] Mackin SR Desai P Whitener BM Karl CE Liu M Baric RS. Fc-γR-dependent antibody effector functions are required for vaccine-mediated protection against antigen-shifted variants of SARS-CoV-2. Nat Microbiol (2023) 8:569–80. doi: 10.1038/s41564-023-01359-1 PMC10797606 37012355

[57] Pai M Behr MA Dowdy D Dheda K Divangahi M Boehme CC. Tuberculosis. Nat Rev Dis Primers (2016) 2:16076. doi: 10.1038/nrdp.2016.76 27784885

[58] Reece ST Kaufmann SHE. Host defenses to intracellular bacteria. In: Rich RR Fleisher TA Harry W. Schroeder J Weyand CM Corry DB Puck JM, editors. Clinical immunology. Principles and practice. Elsevier (2023). p. 331–46.

[59] Dorhoi A Kaufmann SHE. Pathology and immune reactivity: understanding multidimensionality in pulmonary tuberculosis. Semin Immunopathol (2016) 38:153–66. doi: 10.1007/s00281-015-0531-3 26438324

[60] Cohen SB Gern BH Urdahl KB. The tuberculous granuloma and preexisting immunity. Annu Rev Immunol (2022) 40:589–614. doi: 10.1146/annurev-immunol-093019-125148 35130029

[61] Lin PL Ford CB Coleman MT Myers AJ Gawande R Ioerger T. Sterilization of granulomas is common in active and latent tuberculosis despite within-host variability in bacterial killing. Nat Med (2014) 20:75–9. doi: 10.1038/nm.3412 PMC3947310 24336248

[62] Koeken V Verrall AJ Netea MG Hill PC van Crevel R. Trained innate immunity and resistance to Mycobacterium tuberculosis infection. Clin Microbiol Infect (2019) 25:1468–72. doi: 10.1016/j.cmi.2019.02.015 30807849

[63] Abebe F. Immunological basis of early clearance of Mycobacterium tuberculosis infection: the role of natural killer cells. Clin Exp Immunol (2021) 204:32–40. doi: 10.1111/cei.13565 33315236 PMC7944356

[64] Flynn JL Chan J. Immune cell interactions in tuberculosis. Cell (2022) 185:4682–702. doi: 10.1016/j.cell.2022.10.025 PMC12162144 36493751

[65] Kim H Shin SJ. Pathological and protective roles of dendritic cells in Mycobacterium tuberculosis infection: Interaction between host immune responses and pathogen evasion. Front Cell Infect Microbiol (2022) 12:891878. doi: 10.3389/fcimb.2022.891878 35967869 PMC9366614

[66] Ganchua SKC White AG Klein EC Flynn JL. Lymph nodes—The neglected battlefield in tuberculosis. PloS Pathog (2020) 16:e1008632. doi: 10.1371/journal.ppat.1008632 32790739 PMC7425845

[67] Ottenhoff THM Kaufmann SHE. Vaccines against tuberculosis: where are we and where do we need to go? PloS Pathog (2012) 8:e1002607. doi: 10.1371/journal.ppat.1002607 22589713 PMC3349743

[68] Gupta RK Lawn SD Bekker LG Caldwell J Kaplan R Wood R. Impact of human immunodeficiency virus and CD4 count on tuberculosis diagnosis: analysis of city-wide data from Cape Town, South Africa. Int J Tuberc Lung Dis (2013) 17:1014–22. doi: 10.5588/ijtld.13.0032 PMC3990260 23827024

[69] Lawn SD Myer L Edwards D Bekker LG Wood R. Short-term and long-term risk of tuberculosis associated with CD4 cell recovery during antiretroviral therapy in South Africa. AIDS (2009) 23:1717–25. doi: 10.1097/QAD.0b013e32832d3b6d PMC3801095 19461502

[70] Dorhoi A Kaufmann SHE. Tumor necrosis factor alpha in mycobacterial infection. Semin Immunol (2014) 26:203–9. doi: 10.1016/j.smim.2014.04.003 24819298

[71] Sakai S Mayer-Barber KD Barber DL. Defining features of protective CD4 T cell responses to Mycobacterium tuberculosis. Cur Opin Immunol (2014) 29:137–42. doi: 10.1016/j.coi.2014.06.003 PMC4122329 25000593

[72] Keane J Gershon S Wise RP Mirabile-Levens E Kasznica J Schwieterman WD. Tuberculosis associated with infliximab, a tumor necrosis factor α-neutralizing agent. N Engl J Med (2001) 345:1098–104. doi: 10.1056/NEJMoa011110 11596589

[73] Ogongo P Tezera LB Ardain A Nhamoyebonde S Ramsuran D Singh A. Tissue-resident-like CD4+ T cells secreting IL-17 control Mycobacterium tuberculosis in the human lung. J Clin Invest (2021) 131:e142014. doi: 10.1172/JCI142014 33848273 PMC8121523

[74] Coulter F Parrish A Manning D Kampmann B Mendy J Garand M. IL-17 production from T helper 17, mucosal-associated invariant T, and gammadelta cells in tuberculosis infection and disease. Front Immunol (2017) 8:1252. doi: 10.3389/fimmu.2017.01252 29075255 PMC5641565

[75] Noschka R Wondany F Kizilsavas G Weil T Weidinger G Walther P. Gran1: A Granulysin-Derived Peptide with Potent Activity against Intracellular Mycobacterium tuberculosis. Int J Mol Sci (2021) 22:8392. doi: 10.3390/ijms22168392 34445098 PMC8395039

[76] Stenger S Hanson DA Teitelbaum R Dewan P Niazi KR Froelich CJ. An antimicrobial activity of cytolytic T cells mediated by granulysin. Science (1998) 282:121–5. doi: 10.1126/science.282.5386.121 9756476

[77] Woodworth JS Wu Y Behar SM. Mycobacterium tuberculosis-specific CD8+ T cells require perforin to kill target cells and provide protection *in vivo*1. J Immunol (2008) 181:8595–603. doi: 10.4049/jimmunol.181.12.8595 PMC3133658 19050279

[78] Napier RJ Adams EJ Gold MC Lewinsohn DM. The role of mucosal associated invariant T cells in antimicrobial immunity. Front Immunol (2015) 6:344. doi: 10.3389/fimmu.2015.00344 26217338 PMC4492155

[79] Kaufmann SH. Future vaccination strategies against tuberculosis: thinking outside the box. Immunity (2010) 33:567–77. doi: 10.1016/j.immuni.2010.09.015 21029966

[80] Kaufmann SHE. γ/δ and other unconventional T lymphocytes: What do they see and what do they do? Proc Natl Acad Sci USA (1996) 93:2272–9. doi: 10.1073/pnas.93.6.2272 PMC39785 8637862

[81] La Manna MP Orlando V Tamburini B Badami GD Dieli F Caccamo N. Harnessing unconventional T cells for immunotherapy of tuberculosis. Front Immunol (2020) 11:2107. doi: 10.3389/fimmu.2020.02107 33013888 PMC7497315

[82] Lockhart E Green AM Flynn JL. IL-17 Production Is Dominated by γδ T Cells rather than CD4 T Cells during Mycobacterium tuberculosis Infection1. J Immunol (2006) 177:4662–9. doi: 10.4049/jimmunol.177.7.4662 16982905

[83] Ogongo P Steyn AJC Karim F Dullabh KJ Awala I Madansein R. Differential skewing of donor-unrestricted and γδ T cell repertoires in tuberculosis-infected human lungs. J Clin Invest (2020) 130:214–30. doi: 10.1172/JCI130711 PMC6934215 31763997

[84] Van Rhijn I Kasmar A de Jong A Gras S Bhati M Doorenspleet ME. A conserved human T cell population targets mycobacterial antigens presented by CD1b. Nat Immunol (2013) 14:706–13. doi: 10.1038/ni.2630 PMC3723453 23727893

[85] Gold MC Napier RJ Lewinsohn DM. MR1-restricted mucosal associated invariant T (MAIT) cells in the immune response to Mycobacterium tuberculosis. Immunol Rev (2015) 264:154–66. doi: 10.1111/imr.12271 PMC4339229 25703558

[86] Sakai S Kauffman KD Oh S Nelson CE Barry CE Barber DL. MAIT cell-directed therapy of Mycobacterium tuberculosis infection. Mucosal Immunol (2021) 14:199–208. doi: 10.1038/s41385-020-0332-4 32811991 PMC7790750

[87] Ruibal P Voogd L Joosten SA Ottenhoff THM. The role of donor-unrestricted T-cells, innate lymphoid cells, and NK cells in anti-mycobacterial immunity. Immunol Rev (2021) 301:30–47. doi: 10.1111/imr.12948 33529407 PMC8154655

[88] Korchagina AA Koroleva E Tumanov AV. Innate lymphoid cells in response to intracellular pathogens: protection versus immunopathology. Front Cell Infect Microbiol (2021) 11:775554. doi: 10.3389/fcimb.2021.775554 34938670 PMC8685334

[89] Seo GY Giles DA Kronenberg M. The role of innate lymphoid cells in response to microbes at mucosal surfaces. Mucosal Immunol (2020) 13:399–412. doi: 10.1038/s41385-020-0265-y 32047273 PMC7186215

[90] Fillatreau S. B cells and their cytokine activities implications in human diseases. Clin Immunol (2018) 186:26–31. doi: 10.1016/j.clim.2017.07.020 28736271 PMC5844600

[91] Maglione PJ Xu J Chan J. B Cells Moderate Inflammatory Progression and Enhance Bacterial Containment upon Pulmonary Challenge with Mycobacterium tuberculosis1. J Immunol (2007) 178:7222–34. doi: 10.4049/jimmunol.178.11.7222 17513771

[92] Chan J Mehta S Bharrhan S Chen Y Achkar JM Casadevall A. The role of B cells and humoral immunity in Mycobacterium tuberculosis infection. Semin Immunol (2014) 26:588–600. doi: 10.1016/j.smim.2014.10.005 25458990 PMC4314354

[93] Lu LL Chung AW Rosebrock TR Ghebremichael M Yu WH Grace PS. A functional role for antibodies in tuberculosis. Cell (2016) 167:433–443.e14. doi: 10.1016/j.cell.2016.08.072 27667685 PMC5526202

[94] Maglione PJ Xu J Casadevall A Chan J. Fcγ Receptors Regulate Immune Activation and Susceptibility during Mycobacterium tuberculosis Infection1. J Immunol (2008) 180:3329–38. doi: 10.4049/jimmunol.180.5.3329 18292558

[95] Wang TT Ravetch JV. Functional diversification of IgGs through Fc glycosylation. J Clin Invest (2019) 129:3492–8. doi: 10.1172/JCI130029 PMC6715372 31478910

[96] Carpenter SM Lu LL. Leveraging antibody, B cell and fc receptor interactions to understand heterogeneous immune responses in tuberculosis. Front Immunol (2022) 13:830482. doi: 10.3389/fimmu.2022.830482 35371092 PMC8968866

[97] Armstrong JA Hart PD. Response of cultured macrophages to Mycobacterium tuberculosis, with observations on fusion of lysosomes with phagosomes. J Exp Med (1971) 134:713–40. doi: 10.1084/jem.134.3.713 PMC2139093 15776571

[98] Armstrong JA Hart PD. Phagosome-lysosome interactions in cultured macrophages infected with virulent tubercle bacilli. Reversal of the usual nonfusion pattern and observations on bacterial survival. J Exp Med (1975) 142:1–16. doi: 10.1084/jem.142.1.1 807671 PMC2189870

[99] Gideon HP Phuah J Myers AJ Bryson BD Rodgers MA Coleman MT. Variability in tuberculosis granuloma T cell responses exists, but a balance of pro- and anti-inflammatory cytokines is associated with sterilization. PloS Pathog (2015) 11:e1004603. doi: 10.1371/journal.ppat.1004603 25611466 PMC4303275

[100] Kaufmann SH Dorhoi A. Inflammation in tuberculosis: interactions, imbalances and interventions. Curr Opin Immunol (2013) 25:441–9. doi: 10.1016/j.coi.2013.05.005 23725875

[101] Simmons JD Stein CM Seshadri C Campo M Alter G Fortune S. Immunological mechanisms of human resistance to persistent Mycobacterium tuberculosis infection. Nat Rev Immunol (2018) 18:575–89. doi: 10.1038/s41577-018-0025-3 PMC6278832 29895826

[102] Drain PK Bajema KL Dowdy D Dheda K Naidoo K Schumacher SG. Incipient and subclinical tuberculosis: a clinical review of early stages and progression of infection. Clin Microbiol Rev (2018) 31:e00021–18. doi: 10.1128/cmr.00021-18 PMC6148193 30021818

[103] Dorhoi A Reece ST Kaufmann SH. For better or for worse: the immune response against *Mycobacterium tuberculosis* balances pathology and protection. Immunol Rev (2011) 240:235–51. doi: 10.1111/j.1600-065X.2010.00994.x 21349097

[104] Reece ST Kaufmann SH. Floating between the poles of pathology and protection: can we pin down the granuloma in tuberculosis? Curr. Opin Microbiol (2012) 15:63–70. doi: 10.1016/j.mib.2011.10.006 22074861

[105] Kaushal D Foreman TW Gautam US Alvarez X Adekambi T Rangel-Moreno J. Mucosal vaccination with attenuated Mycobacterium tuberculosis induces strong central memory responses and protects against tuberculosis. Nat Commun (2015) 6:8533. doi: 10.1038/ncomms9533 26460802 PMC4608260

[106] Kok L Masopust D Schumacher TN. The precursors of CD8+ tissue resident memory T cells: from lymphoid organs to infected tissues. Nat Rev Immunol (2022) 22:283–93. doi: 10.1038/s41577-021-00590-3 PMC8415193 34480118

[107] Yenyuwadee S Sanchez-Trincado Lopez JL Shah R Rosato PC Boussiotis VA. The evolving role of tissue-resident memory T cells in infections and cancer. Sci Adv (2022) 8:eabo5871. doi: 10.1126/sciadv.abo5871 35977028 PMC9385156

[108] Perdomo C Zedler U Kühl AA Lozza L Saikali P Sander LE. Mucosal BCG vaccination induces protective lung-resident memory T cell populations against tuberculosis. mBio (2016) 7:e01686–16. doi: 10.1128/mBio.01686-16 PMC5120139 27879332

[109] Swanson RV Gupta A Foreman TW Lu L Choreno-Parra JA Mbandi SK. Antigen-specific B cells direct T follicular-like helper cells into lymphoid follicles to mediate Mycobacterium tuberculosis control. Nat Immunol (2023) 24:855–68. doi: 10.1038/s41590-023-01476-3 PMC11133959 37012543

[110] Ulrichs T Kosmiadi GA Trusov V Jörg S Pradl L Titukhina M. Human tuberculous granulomas induce peripheral lymphoid follicle-like structures to orchestrate local host defence in the lung. J Pathol (2004) 204:217–28. doi: 10.1002/path.1628 15376257

[111] Knaul JK Jorg S Oberbeck-Mueller D Heinemann E Scheuermann L Brinkmann V. Lung-residing myeloid-derived suppressors display dual functionality in murine pulmonary tuberculosis. Am J Respir Crit Care Med (2014) 190:1053–66. doi: 10.1164/rccm.201405-0828OC 25275852

[112] Dorhoi A Kotzé LA Berzofsky JA Sui Y Gabrilovich DI Garg A. Therapies for tuberculosis and AIDS: myeloid-derived suppressor cells in focus. J Clin Invest (2020) 130:2789–99. doi: 10.1172/JCI136288 PMC7260010 32420917

[113] Ahmed A Vyakarnam A. Emerging patterns of regulatory T cell function in tuberculosis. Clin Exp Immunol (2020) 202:273–87. doi: 10.1111/cei.13488 PMC7670141 32639588

[114] Barber DL Sakai S KudChadkar RR Fling SP Day TA Vergara JA. Tuberculosis following PD-1 blockade for cancer immunotherapy. Sci Transl Med (2019) 11:eaat2702. doi: 10.1126/scitranslmed.aat2702 30651320 PMC7372940

[115] Jayaraman P Jacques MK Zhu C Steblenko KM Stowell BL Madi A. TIM3 Mediates T Cell Exhaustion during Mycobacterium tuberculosis Infection. PloS Pathog (2016) 12:e1005490. doi: 10.1371/journal.ppat.1005490 26967901 PMC4788425

[116] Tezera LB Bielecka MK Ogongo P Walker NF Ellis M Garay-Baquero DJ. Anti-PD-1 immunotherapy leads to tuberculosis reactivation *via* dysregulation of TNF-α. eLife (2020) 9:e52668. doi: 10.7554/eLife.52668 32091388 PMC7058383

[117] Gengenbacher M Kaufmann SHE. *Mycobacterium tuberculosis*: success through dormancy. FEMS Microbiol Rev (2012) 36:514–32. doi: 10.1111/j.1574-6976.2012.00331.x PMC3319523 22320122

[118] Lewis K. Persister cells. Annu Rev Microbiol (2010) 64:357–72. doi: 10.1146/annurev.micro.112408.134306 20528688

[119] Kaufmann SHE. The TB vaccine development pipeline: present and future priorities and challenges for research and innovation. In: Migliori GB Raviglione ME, editors. Essential tuberculosis. Switzerland: Springer Nature (2021). p. 395–405.

[120] Hanthamrongwit J Aruvornlop P Saelee C Wanta N Poneksawat P Soe PT. Peptide microarray-based identification of dormancy-associated Mycobacterium tuberculosis antigens inducing immune responses among latent tuberculosis infection individuals in Thailand. Sci Rep (2023) 13:6978. doi: 10.1038/s41598-023-34307-4 37117690 PMC10141872

[121] Behr MA Kaufmann E Duffin J Edelstein PH Ramakrishnan L. Latent tuberculosis: two centuries of confusion. Am J Respir Crit Care Med (2021) 204:142–8. doi: 10.1164/rccm.202011-4239PP PMC8650795 33761302

[122] Zak DE Penn-Nicholson A Scriba TJ Thompson E Suliman S Amon LM. A blood RNA signature for tuberculosis disease risk: a prospective cohort study. Lancet (2016) 387:2312–22. doi: 10.1016/S0140-6736(15)01316-1 PMC5392204 27017310

[123] Berry MP Graham CM McNab FW Xu Z Bloch SA Oni T. An interferon-inducible neutrophil-driven blood transcriptional signature in human tuberculosis. Nature (2010) 466:973–7. doi: 10.1038/nature09247 PMC3492754 20725040

[124] Penn-Nicholson A Mbandi SK Thompson E Mendelsohn SC Suliman S Chegou NN. RISK6, a 6-gene transcriptomic signature of TB disease risk, diagnosis and treatment response. Sci Rep (2020) 10:8629. doi: 10.1038/s41598-020-65043-8 32451443 PMC7248089

[125] Suliman S. BCG: From veins to correlates. Cell Host Microbe (2023) 31:921–3. doi: 10.1016/j.chom.2023.05.021 37321176

[126] Weiner J Maertzdorf J Sutherland JS Duffy FJ Thompson E Suliman S. Metabolite changes in blood predict the onset of tuberculosis. Nat Commun (2018) 9:5208. doi: 10.1038/s41467-018-07635-7 30523338 PMC6283869

[127] Diel R Goletti D Ferrara G Bothamley G Cirillo D Kampmann B. Interferon-gamma release assays for the diagnosis of latent Mycobacterium tuberculosis infection: a systematic review and meta-analysis. Eur Respir J (2011) 37:88–99. doi: 10.1183/09031936.00115110 21030451

[128] Pai M Zwerling A Menzies D. Systematic review: T-cell-based assays for the diagnosis of latent tuberculosis infection: an update. Ann Intern Med (2008) 149:177–84. doi: 10.7326/0003-4819-149-3-200808050-00241 PMC2951987 18593687

[129] Comstock GW Livesay VT Woolpert SF. The prognosis of a positive tuberculin reaction in childhood and adolescence. Am J Epidemiol (1974) 99:131–8. doi: 10.1093/oxfordjournals.aje.a121593 4810628

[130] Eisenhut M Paranjothy S Abubakar I Bracebridge S Lilley M Mulla R. BCG vaccination reduces risk of infection with Mycobacterium tuberculosis as detected by gamma interferon release assay. Vaccine (2009) 27:6116–20. doi: 10.1016/j.vaccine.2009.08.031 19715782

[131] Kaipilyawar V Salgame P. Infection resisters: targets of new research for uncovering natural protective immunity against Mycobacterium tuberculosis. F1000Research (2019) 8:1698. doi: 10.12688/f1000research.19805.1 PMC6774050 31602294

[132] Stein CM Nsereko M Malone LL Okware B Kisingo H Nalukwago S. Long-term stability of resistance to latent mycobacterium tuberculosis infection in highly exposed tuberculosis household contacts in Kampala, Uganda. Clin Infect Dis (2018) 68:1705–12. doi: 10.1093/cid/ciy751 PMC6495009 30165605

[133] Medawar L Tukiman HM Mbayo G Donkor S Owolabi O Sutherland JS. Analysis of cellular and soluble profiles in QuantiFERON nonconverters, converters, and reverters in the Gambia. Immun Inflamm Dis (2019) 7:260–70. doi: 10.1002/iid3.269 PMC6842814 31430056

[134] Garcia-Basteiro AL White RG Tait D Schmidt AC Rangaka MX Quaife M. End-point definition and trial design to advance tuberculosis vaccine development. Eur Respir Rev (2022) 31:220044. doi: 10.1183/16000617.0044-2022 35675923 PMC9488660

[135] Mpande CAM Steigler P Lloyd T Rozot V Mosito B Schreuder C. Mycobacterium tuberculosis-specific T cell functional, memory, and activation profiles in quantiFERON-reverters are consistent with controlled infection. Front Immunol (2021) 12:712480. doi: 10.3389/fimmu.2021.712480 34526988 PMC8435731

[136] Dinkele R Gessner S McKerry A Leonard B Seldon R Koch AS. Capture and visualization of live Mycobacterium tuberculosis bacilli from tuberculosis patient bioaerosols. PloS Pathog (2021) 17:e1009262. doi: 10.1371/journal.ppat.1009262 33524021 PMC7877778

[137] Nguyen HV Tiemersma E Nguyen NV Nguyen HB Cobelens F. Disease transmission by patients with subclinical tuberculosis. Clin Infect Dis (2023) 76:2000–6. doi: 10.1093/cid/ciad027 PMC10249982 36660850

[138] Patterson B Bryden W Call C McKerry A Leonard B Seldon R. Cough-independent production of viable Mycobacterium tuberculosis in bioaerosol. Tuberculosis (2021) 126:102038. doi: 10.1016/j.tube.2020.102038 33316737

[139] Ryckman TS Dowdy DW Kendall EA. Infectious and clinical tuberculosis trajectories: Bayesian modeling with case finding implications. Proc Natl Acad Sci U.S.A. (2022) 119:e2211045119. doi: 10.1073/pnas.2211045119 36534797 PMC9907102

[140] Bouzeyen R Javid B. Therapeutic vaccines for tuberculosis: an overview. Front Immunol (2022) 13:878471. doi: 10.3389/fimmu.2022.878471 35812462 PMC9263712

[141] Nemes E Fiore-Gartland A Boggiano C Coccia M D'Souza P Gilbert P. The quest for vaccine-induced immune correlates of protection against tuberculosis. Vaccine Insights (2022) 1:165–81. doi: 10.18609/vac/2022.027 PMC10117634 37091190

[142] Sadoff JC Wittes J. Correlates, surrogates, and vaccines. J Infect Dis (2007) 196:1279–81. doi: 10.1086/522432 17922389

[143] World Health Organization. Correlates of vaccine-induced protection: methods and implications (2013). Available at: https://apps.who.int/iris/handle/10665/84288.

[144] Britto C Alter G. The next frontier in vaccine design: blending immune correlates of protection into rational vaccine design. Curr Opin Immunol (2022) 78:102234. doi: 10.1016/j.coi.2022.102234 35973352 PMC9612370

[145] Plotkin SA. Correlates of protection induced by vaccination. Clin Vaccine Immunol (2010) 17:1055–65. doi: 10.1128/cvi.00131-10 PMC2897268 20463105

[146] Plotkin SA Gilbert PB. Nomenclature for immune correlates of protection after vaccination. Clin Infect Dis (2012) 54:1615–7. doi: 10.1093/cid/cis238 PMC3348952 22437237

[147] Bitencourt J Peralta-Álvarez MP Wilkie M Jacobs A Wright D Salman Almujri S. Induction of functional specific antibodies, igG-secreting plasmablasts and memory B cells following BCG vaccination. Front Immunol (2021) 12:798207. doi: 10.3389/fimmu.2021.798207 35069580 PMC8767055

[148] Kawahara JY Irvine EB Alter G. A case for antibodies as mechanistic correlates of immunity in tuberculosis. Front Immunol (2019) 10:996. doi: 10.3389/fimmu.2019.00996 31143177 PMC6521799

[149] Nziza N Cizmeci D Davies L Irvine EB Jung W Fenderson BA. Defining discriminatory antibody fingerprints in active and latent tuberculosis. Front Immunol (2022) 13:856906. doi: 10.3389/fimmu.2022.856906 35514994 PMC9066635

[150] Musvosvi M Huang H Wang C Xia Q Rozot V Krishnan A. T cell receptor repertoires associated with control and disease progression following Mycobacterium tuberculosis infection. Nat Med (2023) 29:258–69. doi: 10.1038/s41591-022-02110-9 PMC9873565 36604540

[151] Kaufmann SH Evans TG Hanekom WA. Tuberculosis vaccines: time for a global strategy. Sci Transl Med (2015) 7:276fs8. doi: 10.1126/scitranslmed.aaa4730 25717094

[152] Kaufmann SHE. Vaccination against tuberculosis: revamping BCG by molecular genetics guided by immunology. Front Immunol (2020) 11:316. doi: 10.3389/fimmu.2020.00316 32174919 PMC7056705

[153] Calmette A Guérin C Boquet A Négre L. La vaccination préventive contre la tuberculose par le "BCG". Masson, Paris: Masson et Cie (1927). p. 250.

[154] Trunz BB Fine P Dye C. Effect of BCG vaccination on childhood tuberculous meningitis and miliary tuberculosis worldwide: a meta-analysis and assessment of cost-effectiveness. Lancet (2006) 367:1173–80. doi: 10.1016/s0140-6736(06)68507-3 16616560

[155] Nemes E Geldenhuys H Rozot V Rutkowski KT Ratangee F Bilek N. Prevention of M. tuberculosis infection with H4:IC31 vaccine or BCG revaccination. N Engl J Med (2018) 379:138–49. doi: 10.1056/NEJMoa1714021 PMC5937161 29996082

[156] Darrah PA Zeppa JJ Maiello P Hackney JA Wadsworth MH Hughes TK. Prevention of tuberculosis in macaques after intravenous BCG immunization. Nature (2020) 577:95–102. doi: 10.1038/s41586-019-1817-8 31894150 PMC7015856

[157] Darrah PA Zeppa JJ Wang C Irvine EB Bucsan AN Rodgers MA. Airway T cells are a correlate of i.v. Bacille Calmette-Guerin-mediated protection against tuberculosis in rhesus macaques. Cell Host Microbe (2023) 31:962–977.e8. doi: 10.1016/j.chom.2023.05.006 37267955 PMC10355173

[158] Liu YE Darrah PA Zeppa JJ Kamath M Laboune F Douek DC. Blood transcriptional correlates of BCG-induced protection against tuberculosis in rhesus macaques. Cell Rep Med (2023) 4:101096. doi: 10.1016/j.xcrm.2023.101096 37390827 PMC10394165

[159] Jenum S Tonby K Rueegg CS Rühwald M Kristiansen MP Bang P. A Phase I/II randomized trial of H56:IC31 vaccination and adjunctive cyclooxygenase-2-inhibitor treatment in tuberculosis patients. Nat Commun (2021) 12:6774. doi: 10.1038/s41467-021-27029-6 34811370 PMC8608791

[160] Sagawa ZK Goman C Frevol A Blazevic A Tennant J Fisher B. Safety and immunogenicity of a thermostable ID93 + GLA-SE tuberculosis vaccine candidate in healthy adults. Nat Commun (2023) 14:1138. doi: 10.1038/s41467-023-36789-2 36878897 PMC9988862

[161] Tkachuk AP Bykonia EN Popova LI Kleymenov DA Semashko MA Chulanov VP. Safety and immunogenicity of the gamTBvac, the recombinant subunit tuberculosis vaccine candidate: A phase II, multi-center, double-blind, randomized, placebo-controlled study. Vaccines (2020) 8:652. doi: 10.3390/vaccines8040652 33153191 PMC7712213

[162] Tait DR Hatherill M van der Meeren O Ginsberg AM Van Brakel E Salaun B. Final analysis of a trial of M72/AS01E vaccine to prevent tuberculosis. N Engl J Med (2019) 381:2429–39. doi: 10.1056/NEJMoa1909953 31661198

[163] Van Der Meeren O Hatherill M Nduba V Wilkinson RJ Muyoyeta M Van Brakel E. Phase 2b controlled trial of M72/AS01E vaccine to prevent tuberculosis. N Engl J Med (2018) 379:1621–34. doi: 10.1056/NEJMoa1803484 PMC6151253 30280651

[164] Jeyanathan M Fritz DK Afkhami S Aguirre E Howie KJ Zganiacz A. but not intramuscular injection, of adenovirus-vectored tuberculosis vaccine induces respiratory-mucosal immunity in humans. JCI Insight (2022) 7:e155655. doi: 10.1172/jci.insight.155655 34990408 PMC8855837

[165] Wilkie M Satti I Minhinnick A Harris S Riste M Ramon RL. A phase I trial evaluating the safety and immunogenicity of a candidate tuberculosis vaccination regimen, ChAdOx1 85A prime – MVA85A boost in healthy UK adults. Vaccine (2020) 38:779–89. doi: 10.1016/j.vaccine.2019.10.102 PMC6985898 31735500

[166] McShane H Pathan AA Sander CR Keating SM Gilbert SC Huygen K. Recombinant modified vaccinia virus Ankara expressing antigen 85A boosts BCG-primed and naturally acquired antimycobacterial immunity in humans. Nat Med (2004) 10:1240–4. doi: 10.1038/nm1128 15502839

[167] Ndiaye BP Thienemann F Ota M Landry BS Camara M Dieye S. Safety, immunogenicity, and efficacy of the candidate tuberculosis vaccine MVA85A in healthy adults infected with HIV-1: a randomised, placebo-controlled, phase 2 trial. Lancet Respir Med (2015) 3:190–200. doi: 10.1016/S2213-2600(15)00037-5 25726088 PMC4648060

[168] Tameris MD Hatherill M Landry BS Scriba TJ Snowden MA Lockhart S. Safety and efficacy of MVA85A, a new tuberculosis vaccine, in infants previously vaccinated with BCG: a randomised, placebo-controlled phase 2b trial. Lancet (2013) 381:1021–8. doi: 10.1016/S0140-6736(13)60177-4 PMC5424647 23391465

[169] Cardona PJ. RUTI: a new chance to shorten the treatment of latent tuberculosis infection. Tuberculosis (2006) 86:273–89. doi: 10.1016/j.tube.2006.01.024 16545981

[170] Munseri P Said J Amour M Magohe A Matee M Rees CA. DAR-901 vaccine for the prevention of infection with Mycobacterium tuberculosis among BCG-immunized adolescents in Tanzania: A randomized controlled, double-blind phase 2b trial. Vaccine (2020) 38:7239–45. doi: 10.1016/j.vaccine.2020.09.055 33004239

[171] Sharma SK Katoch K Sarin R Balambal R Kumar Jain N Patel N. Efficacy and Safety of Mycobacterium indicus pranii as an adjunct therapy in Category II pulmonary tuberculosis in a randomized trial. Sci Rep (2017) 7:3354. doi: 10.1038/s41598-017-03514-1 28611374 PMC5469738

[172] Martín C Marinova D Aguiló N Gonzalo-Asensio J. MTBVAC, a live TB vaccine poised to initiate efficacy trials 100 years after BCG. Vaccine (2021) 39:7277–85. doi: 10.1016/j.vaccine.2021.06.049 34238608

[173] Grode L Seiler P Baumann S Hess J Brinkmann V Nasser Eddine A. Increased vaccine efficacy against tuberculosis of recombinant *Mycobacterium bovis* bacille Calmette-Guérin mutants that secrete listeriolysin. J Clin Invest (2005) 115:2472–9. doi: 10.1172/JCI24617 PMC1187936 16110326

[174] Cole ST Brosch R Parkhill J Garnier T Churcher C Harris D. Deciphering the biology of Mycobacterium tuberculosis from the complete genome sequence. Nature (1998) 393:537–44. doi: 10.1038/31159 9634230

[175] Treatment Action Group (TAG). Stop TB partnership. Tuberculosis research funding trends, 2005-2020 (2021). Available at: https://www.treatmentactiongroup.org/wp-content/uploads/2021/12/tb_funding_2021.pdf.

[176] World Health Organization. An investment case for new tuberculosis vaccines (2022). Available at: https://apps.who.int/iris/rest/bitstreams/1485059/retrieve.

[177] Boseley S. ‘Gamechanging’ TB vaccine within reach after $500m pledge to run final trials. United Kingdom: The Guardian (2023). Available at: https://www.theguardian.com/global-development/2023/jun/28/bill-gates-wellcome-tb-vaccine-within-reach-after-500m-pledge-to-run-final-trials.

[178] ITCR/ICMR. Need for effective TB vaccines. Available at: https://itrc.icmr.org.in/our-work/thematic-areas/vaccines.

[179] Kaufmann SHE. Highly affordable vaccines are critical for our continued efforts to reduce global childhood mortality. Hum Vaccin Immunother (2019) 15:2660–5. doi: 10.1080/21645515.2019.1605817 PMC6930051 30973039

[180] GAVI The Vaccine Alliance. COVAX. Available at: https://www.gavi.org/covax-facility.

[181] World Health Organization. COVAX. Available at: https://www.who.int/initiatives/act-accelerator/covax.

[182] Portnoy A Clark RA Quaife M Weerasuriya CK Mukandavire C Bakker R. The cost and cost-effectiveness of novel tuberculosis vaccines in low- and middle-income countries: A modeling study. PloS Med (2023) 20:e1004155. doi: 10.1371/journal.pmed.1004155 36693081 PMC9873163

[183] United Nations. WHO announces first technology recipients of mRNA vaccine hub with strong support from African and European partners (2022). Available at: https://www.un.org/africarenewal/magazine/who-announces-first-technology-recipients-mrna-vaccine-hub-strong-support-african-and.

[184] Larson HJ Gakidou E Murray CJL. The vaccine-hesitant moment. N Engl J Med (2022) 387:58–65. doi: 10.1056/NEJMra2106441 35767527 PMC9258752

[185] Sorell T Butler J. The politics of covid vaccine hesitancy and opposition. . Polit Q (2022) 93:347–51. doi: 10.1111/1467-923x.13134 PMC9111101 35600736

[186] Singh P Dhalaria P Kashyap S Soni GK Nandi P Ghosh S. Strategies to overcome vaccine hesitancy: a systematic review. Syst Rev (2022) 11:78. doi: 10.1186/s13643-022-01941-4 35473819 PMC9044888

[187] Tuckerman J Kaufman J Danchin M. Effective approaches to combat vaccine hesitancy. Pediatr Infect Dis J (2022) 41:e243–5. doi: 10.1097/inf.0000000000003499 PMC8997018 35213864

[188] World Health Organization. End TB by 2030. Framework for implementing the “End TB strategy” in the african region 2016 - 2020 (2017). Available at: https://apps.who.int/iris/bitstream/handle/10665/259636/TBstrat-eng.pdf?sequence=1.

[189] Stop TB Partnership. The Global Plan to end TB 2023-2030 (2022). Available at: https://www.stoptb.org/file/15583/download.

[190] World Health Organization. The end TB strategy report (2015). Available at: https://www.who.int/publications/i/item/WHO-HTM-TB-2015.19.

[191] World Health Organization. UN General Assembly high-level meeting on tuberculosis (2018). Available at: https://www.who.int/teams/global-tuberculosis-programme/unga-high-level-meeting-on-the-fight-against-tb/unga-high-level-meeting-on-ending-tb.

[192] Stop TB Partnership. TB vaccine clinical pipeline (2023). Available at: https://newtbvaccines.org/tb-vaccine-pipeline/.

[193] Stop TB Partnership. United nations high-level meeting on TB 2023. Available at: https://www.stoptb.org/advocate-to-endtb/united-nations-high-level-meeting-tb.

